# Human Tetherin Exerts Strong Selection Pressure on the HIV-1 Group N Vpu Protein

**DOI:** 10.1371/journal.ppat.1003093

**Published:** 2012-12-20

**Authors:** Daniel Sauter, Daniel Unterweger, Michael Vogl, Shariq M. Usmani, Anke Heigele, Silvia F. Kluge, Elisabeth Hermkes, Markus Moll, Edward Barker, Martine Peeters, Gerald H. Learn, Frederic Bibollet-Ruche, Joëlle V. Fritz, Oliver T. Fackler, Beatrice H. Hahn, Frank Kirchhoff

**Affiliations:** 1 Institute of Molecular Virology, Ulm University Medical Center, Ulm, Germany; 2 Center for Infectious Medicine, Department of Medicine, Karolinska Institutet, Karolinska University Hospital Huddinge, Stockholm, Sweden; 3 Department of Immunology and Microbiology, Rush University Medical Center, Chicago, Illinois, United States of America; 4 Laboratoire Retrovirus UMR145, IRD and Université Montpellier, BP64501, Montpellier, France; 5 Department of Medicine, University of Pennsylvania, Philadelphia, Pennsylvania, United States of America; 6 Department of Infectious Diseases, Virology, University Hospital Heidelberg, Heidelberg, Germany; 7 Department of Microbiology, University of Pennsylvania, Philadelphia, Pennsylvania, United States of America; Vanderbilt University School of Medicine, United States of America

## Abstract

HIV-1 groups M and N emerged within the last century following two independent cross-species transmissions of SIVcpz from chimpanzees to humans. In contrast to pandemic group M strains, HIV-1 group N viruses are exceedingly rare, with only about a dozen infections identified, all but one in individuals from Cameroon. Poor adaptation to the human host may be responsible for this limited spread of HIV-1 group N in the human population. Here, we analyzed the function of Vpu proteins from seven group N strains from Cameroon, the place where this zoonosis originally emerged. We found that these N-Vpus acquired four amino acid substitutions (E15A, V19A and IV25/26LL) in their transmembrane domain (TMD) that allow efficient interaction with human tetherin. However, despite these adaptive changes, most N-Vpus still antagonize human tetherin only poorly and fail to down-modulate CD4, the natural killer (NK) cell ligand NTB-A as well as the lipid-antigen presenting protein CD1d. These functional deficiencies were mapped to amino acid changes in the cytoplasmic domain that disrupt putative adaptor protein binding sites and an otherwise highly conserved ßTrCP-binding DSGxxS motif. As a consequence, N-Vpus exhibited aberrant intracellular localization and/or failed to recruit the ubiquitin-ligase complex to induce tetherin degradation. The only exception was the Vpu of a group N strain recently discovered in France, but originally acquired in Togo, which contained intact cytoplasmic motifs and counteracted tetherin as effectively as the Vpus of pandemic HIV-1 M strains. These results indicate that HIV-1 group N Vpu is under strong host-specific selection pressure and that the acquisition of effective tetherin antagonism may lead to the emergence of viral variants with increased transmission fitness.

## Author Summary

Differences in their degree of adaptation to humans may explain why only one of four ape-derived SIV zoonoses spawned the AIDS pandemic. Specifically, only HIV-1 strains of the pandemic M group evolved a fully functional Vpu that efficiently antagonizes human tetherin and degrades CD4. In comparison, the rare group N viruses gained some anti-tetherin activity but lost the CD4 degradation function. Here, we show that the N-Vpu transmembrane domain has adapted to interact with human tetherin and identified the mutations that enable this interaction. However, we also show that most N-Vpus remain poor tetherin antagonists and fail to reduce the surface expression of CD4, the natural killer cell ligand NTB-A and the lipid-antigen presenting protein CD1d. This is due to mutations in their cytoplasmic region that are associated with aberrant protein localization and impaired interaction with the ubiquitin/proteasome pathway. A remarkable exception is the Vpu of the first HIV-1 N strain known to be transmitted outside of Cameroon, which contains a functional cytoplasmic domain and is a highly effective tetherin antagonist. These data indicate that group N viruses are still adapting to humans and that the acquisition of potent anti-tetherin activity may eventually lead to the emergence of viral variants that exhibit increased transmission fitness.

## Introduction

HIV-1 is the result of at least four independent cross-species transmissions of SIVs from chimpanzees or gorillas to humans [1]. The resulting pathogens, termed HIV-1 groups M, O, N and P, differ greatly in their spread within the human population. The main group M was introduced from a chimpanzee early in the last century and is responsible for the global AIDS epidemic [1]. In contrast, the rare group N, which is also of chimpanzee origin, has thus far only been identified in about a dozen people, all but one from Cameroon [2]–[7]. The remaining two groups O and P are more closely related to SIVgor infecting gorillas [1], [8]. HIV-1 O has infected tens of thousands of individuals, but is geographically restricted to Cameroon and surrounding countries, while group P has only been found in two individuals from Cameroon [8], [9].

Differences in their degree of adaptation are one likely reason for the varying spread of the four groups of HIV-1 within the human population, particularly since all of these ape-to-human transmissions occurred within the past century [1] and because humans are equipped with anti-viral restriction factors that often have to be counteracted by viral proteins in a species-specific manner [10], [11]. One of these restriction factors is tetherin (BST-2 or CD317), which poses a particularly effective barrier to primate lentiviral transmissions [12]. Tetherin is an interferon-induced type 2 integral membrane protein that contains a cytoplasmic N-terminal region, a transmembrane domain (TMD), a coiled-coil extracellular domain, and a C-terminal glycophosphatidylinositol (GPI) anchor [13], [14]. Tetherin inhibits virion release by directly tethering nascent virions to the cell surface [15]–[17]. With the exception of SIVs from certain guenons (i.e., SIVs from greater spot-nosed, mustached and mona monkeys), which use Vpu to counteract tetherin, the great majority of SIVs, including SIVcpz and SIVgor, use their Nef protein to antagonize this restriction factor [18]–[20]. Human tetherin, however, is resistant to Nef due to a five amino acid deletion in its cytoplasmic domain [18]–[20]. Thus, the SIVcpz and SIVgor precursors of HIV-1 were initially unable to antagonize the human tetherin orthologue. During adaptation to humans, group M viruses gained Vpu-mediated anti-tetherin activity [19]. However, this was not the case for HIV-1 O and P strains, whose Vpu failed to acquire this activity. In comparison, group N Vpus gained modest anti-tetherin activity, but concomitantly lost their CD4 degradation function [19], [21]–[23]. Thus, only pandemic group M viruses evolved fully functional Vpus that degrade CD4 and counteract human tetherin with high efficiency.

It is largely unknown which determinants in the Vpu proteins of group N viruses (N-Vpus) are responsible for the modest gain of anti-tetherin activity and the loss of the CD4 degradation function. Moreover, it is unknown whether N-Vpus, like M-Vpus, can reduce the cell surface expression of the natural killer (NK) cell ligand NTB-A and the lipid-antigen presenting protein CD1d, which protect virally infected cells against NK cells and natural killer T (NKT) cells, respectively [24], [25]. To address these questions, we analyzed the Vpu proteins of eight group N viruses, along with a large panel of site-directed mutants and recombinants. We found that group N strains encode Vpus whose transmembrane domains have evolved to interact efficiently with human tetherin. However, our data also show that most N-Vpus are still unable to down-modulate CD4, CD1d and NTB-A, and antagonize tetherin only poorly because they contain disruptive changes in their cytoplasmic region. A notable exception was the Vpu protein from a group N virus recently discovered in a patient with acute symptomatic HIV-1 infection, which contained intact cytoplasmic motifs critical for tetherin antagonism and counteracted this antiviral factor as efficiently as the Vpus of pandemic HIV-1 M strains. This group N virus was discovered in Paris but was most likely acquired in Togo [3], and is thus the first member of the group N lineage to be identified outside of Cameroon. These results indicate that HIV-1 group N Vpu is under strong host-specific selection, which continues to shape its function and may eventually result in the emergence of viral variants with increased transmission fitness.

## Results

### Vpus from Cameroonian group N viruses are poor tetherin antagonists and largely unable to down-modulate CD4, CD1d and NTB-A

Previous studies of three HIV-1 group N strains (YBF30, 2693BA and CK1.62) indicated that their Vpu proteins had gained modest anti-tetherin activity, but lost the ability to degrade CD4 [19]. To determine whether these functional properties were representative of N-Vpus, we analyzed four additional *vpu* alleles from Cameroonian group N strains (Table S1). Using this expanded panel, we investigated previously established Vpu functions, i.e. tetherin antagonism and CD4 down modulation, as well as more recently discovered Vpu activities, i.e., CD1d and NTB-A cell surface modulation. The latter receptors were described to be down-modulated by M-Vpus to facilitate viral immune evasion from NK and NKT cells, respectively [24], [25]. As controls, we tested *vpu* genes from 25 HIV-1 group M strains representing all subtypes, and from ten SIVcpz strains representing both central and eastern chimpanzee virus lineages [19]. With the exception of the U14296 *vpu* gene, which contained a 30 nucleotide deletion in the first α-helical region [6], all others (n = 42) expressed detectable, albeit highly variable, levels of Vpu proteins (Figure S1A) [19].

**Figure 1:**
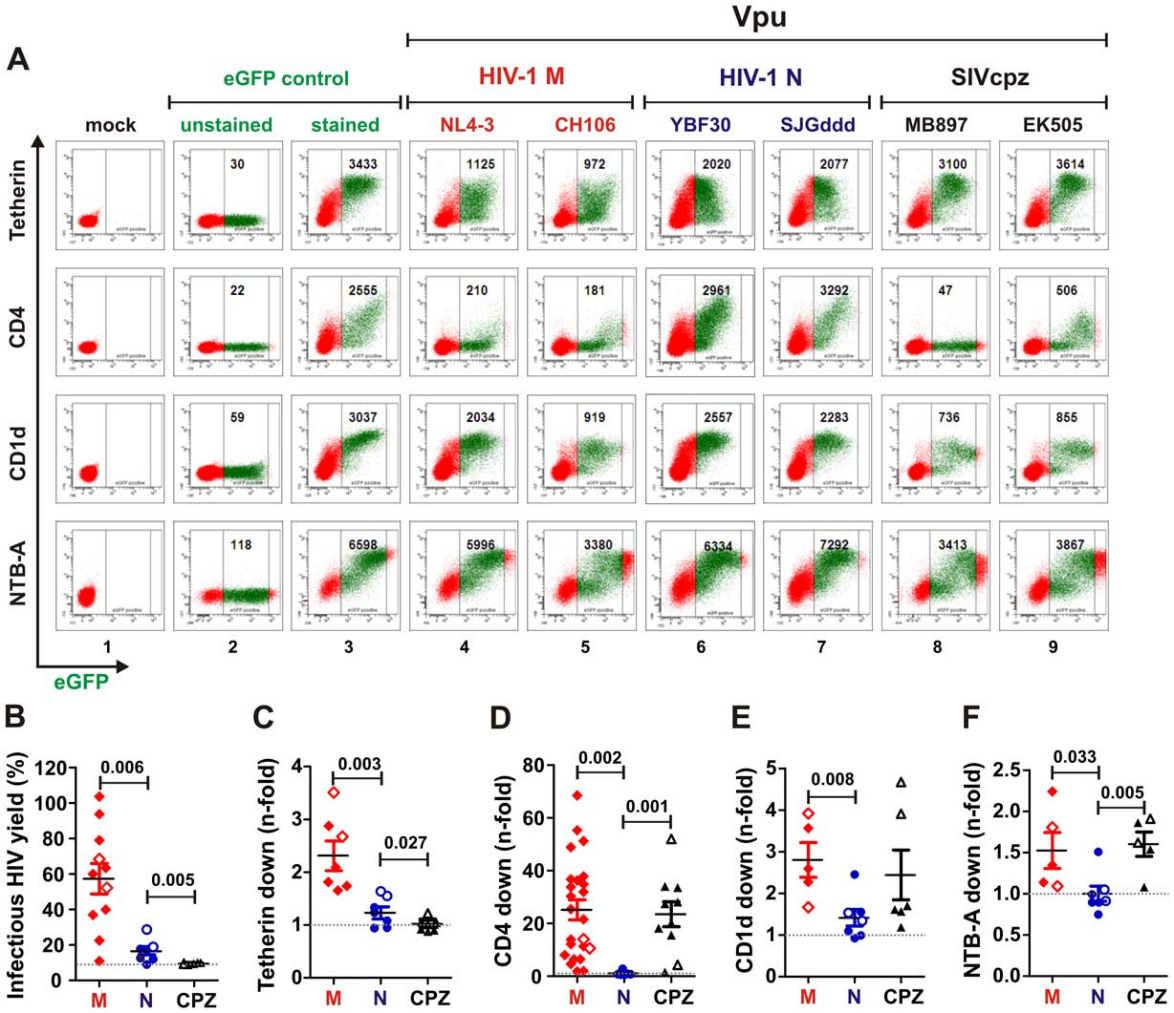
Functional characterization of Vpus from Cameroonian HIV-1 N strains. (**A**) FACS analysis of 293T cells cotransfected with tetherin, CD4, CD1d or NTB-A expression vectors and pCGCG plasmids expressing eGFP alone (lanes 2 and 3) or together with the indicated *vpu* allele (lanes 4 to 9). eGFP expression levels used to calculate receptor downmodulation and the mean fluorescence intensities (MFIs) are indicated. (**B**) Effect of various Vpus on infectious virus release. 293T cells were cotransfected with HIV-1 ΔVpu NL4-3 (2 µg), pCGCG vectors coexpressing eGFP and Vpu (500 ng), and a construct expressing HU tetherin (125 ng). Viral supernatants were obtained two days later and used to measure the quantity of infectious HIV-1 in the culture supernatants by infecting TZM-bl indicator cells. Shown is the infectious virion yield relative to that obtained in the absence of tetherin (100%). The results were confirmed in two independent experiments and each symbol indicates infectious virus yield in the presence of one of the 25 *vpu* alleles analyzed. (**C–F**) Vpu-dependent reduction of (**C**) tetherin, (**D**) CD4, (**E**) CD1d and (**F**) NTB-A surface expression in 293T cells. Shown is the reduction in the levels of receptor cell surface expression relative to those measured in cells transfected with the eGFP only control vector. Each symbol represents n-fold downmodulation of the indicated receptor molecule by one individual *vpu* allele examined. Shown are average values derived from three experiments. *Vpu* alleles derived from HIV-1 M are color coded red, N blue (except for the DJO0131 Vpu highlighted in green) and SIVcpz black; those shown in panel A are indicated by open symbols in panels B to F.

To determine the ability of the various Vpus to modulate receptor surface expression, we transfected 293T cells with vectors co-expressing Vpu and eGFP together with constructs expressing human (HU) tetherin, CD4, CD1d or NTB-A. In the absence of Vpu, all four receptors were expressed at high levels (Figure 1A, panel 3). Co-expression of M-Vpus reduced the cell surface expression of CD4 and (to a lesser extent) of CD1d, NTB-A and tetherin, while co-expression of N-Vpus had little if any effect (Figure 1A, panels 4–7). Quantitative analyses confirmed that N-Vpus were significantly less active than M-Vpus in antagonizing tetherin and unable to down-modulate the cell surface levels of CD4, CD1d and NTB-A (Figure 1B–F). The single exception was the DJO0131 Vpu, which reduced CD4 expression by 2.8-fold and also displayed modest activity in CD1d and NTB-A modulation (Figure 1E, 1F). Similar to M-Vpus, most SIVcpz (CPZ) Vpus down-modulated human CD4 and NTB-A. Furthermore, the Vpus of SIVcpz MB897 and EK505, which represent the closest known genetic relatives of HIV-1 groups M and N viruses, respectively, were also active against CD1d (Figure 1A panels 8 and 9, 1E). The efficiencies of CD1d, NTB-A and CD4 (but not tetherin) down-modulation correlated, suggesting that they are mediated by overlapping domains in Vpu (Figure S1B). Of note, these Vpu functions generally did not correlate with the *in vitro* Vpu expression levels detected by Western blot (Figure S1C). This is in agreement with our previous finding that some Vpu proteins are highly active in functional assays (e.g. the SIVcpz*Pts* TAN3 Vpu in CD4 degradation) but hardly detectable by Western blot analysis [19], possibly because they aggregate or remain associated with the insoluble membrane fraction. In comparison, N-Vpus were generally poorly active although some of them were detectable at high levels.

**Figure 2:**
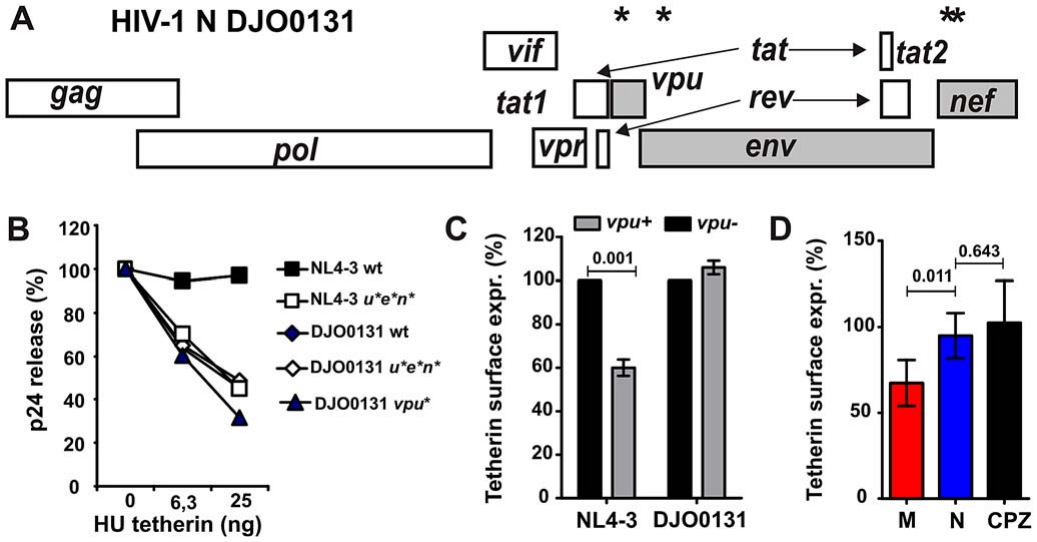
Analysis of N-Vpu function in the context of HIV-1 proviral constructs. (**A**) Location of modifications introduced into a replication competent HIV-1 group N (DJO0131) proviral clone. Asterisks indicate the positions of mutations in the *vpu*, *env* and *nef* genes, respectively. (**B**) 293T cells were cotransfected with the indicated proviral constructs and different quantities of tetherin expression constructs. The levels of p24 released in the culture supernatant were determined as described in the methods section. The results were confirmed in three independent experiments and by measuring the reverse transcriptase activity in the cell culture supernatants. (**C**) PHA-activated PBMCs were transduced with VSV-G pseudotyped wild-type or *vpu*-defective HIV-1 M NL4-3 or N DJO0131 constructs and examined for intracellular p24 capsid antigen and tetherin surface expression five days later. The results in panels C and D show average levels (±SD) of tetherin surface expression in virally infected (p24+) cells relative to uninfected cells (100%) and were derived from three independent experiments with two different PBMC donors in each experiment. (**D**) PHA-stimulated PBMCs were transduced with VSV-G pseudotyped NL4-3 IRES *env* constructs expressing group M (NL4-3, JR-CSF, YU-2), N (YBF30) or SIVcpz (GAB1, MP7) *vpu* alleles and analyzed by flow cytometric analysis as described in panel C.

The data described above suggested that most group N viruses have not evolved an effective antagonist of human tetherin. To confirm the functional inferiority of N-Vpus, we generated an infectious molecular clone of the HIV-1 group N strain DJO0131 [2] and introduced inactivating mutations into the *vpu*, *env* and *nef* genes (Figure 2A). Quantification of virion release in the presence of increasing amounts of human tetherin revealed that DJO0131 Vpu failed to antagonize tetherin (Figure 2B). Importantly, neither Nef nor Env compensated for this lack of anti-tetherin activity, although some primate lentiviruses use these proteins to counteract this restriction factor [11], [12]. In contrast to HIV-1 M NL4-3, the group N DJO0131 virus was also unable to reduce tetherin surface expression in virally infected peripheral blood mononuclear cells (PBMCs) (Figure 2C). The poor anti-tetherin activity and lack of CD4, CD1d and NTB-A function of N-Vpus was confirmed in primary cells using previously described infectious NL4-3-based molecular clones [18] expressing various group M, N and SIVcpz Vpu proteins (Figure 2D). Altogether, these results indicate that N-Vpus are lacking most M-Vpu activities. Unexpectedly, however, N-Vpus were also inferior to CPZ-Vpus in no less than three functions (down-modulation of CD4, CD1d and NTB-A) indicating that human adaptation caused a loss of several functions that were originally present in SIVcpz.

### N-Vpus contain a functional TMD but defective cytoplasmic domains

**Figure 3:**
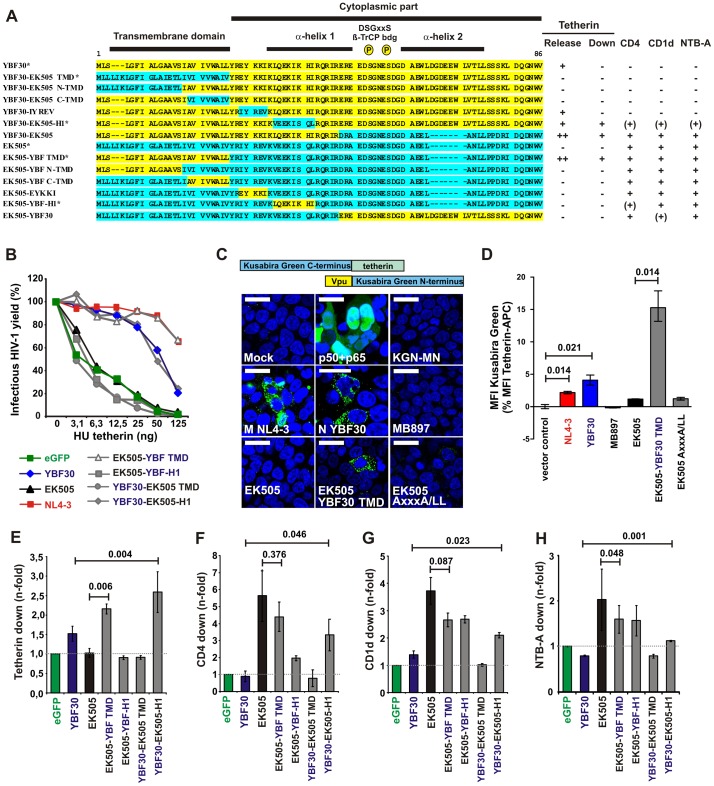
Determinants of functional differences between HIV-1 N and SIVcpz Vpus. (**A**) Chimeras between the YBF30 (yellow) and EK505 (green) Vpu proteins analyzed. The hydrophobic TMD, the α-helical regions and the position of two serine phosphorylation sites are indicated. Dashes indicate gaps introduced to optimize the alignment. The right panel summarizes the functional activity of the respective wild-type or chimeric Vpu proteins. Abbreviations: −, no; (+) low; +, modest and ++ high activity. The asterisks indicate the Vpus shown in panels **B** to **F**. (**B**) Effect of selected wild-type and mutant YBF30 and EK505 Vpu proteins and the NL4-3 Vpu on infectious virus release in the presence of tetherin. Curves represent the average infection values (n = 3) relative to those obtained in the absence of tetherin. (**C**) Interaction between tetherin and Vpu detected by BiFC. 293T cells transfected with plasmids expressing tetherin and the indicated Vpu proteins were examined by confocal microscopy. (**D**) Quantitative BiFC assay. Cells were transfected as described in panel A and analyzed by flow cytometry. Shown is the mean fluorescence intensity (MFI) of Kusabira green relative to the MFI of tetherin (100%). The results represent the means from three independent experiments (±SD). (**E–H**) Efficiencies of down-modulation of (**E**) tetherin, (**F**) CD4, (**G**) CD1d and (**H**) NTB-A cell surface expression in the presence of constructs expressing the indicate Vpu proteins relative to those measured in cells transfected with the eGFP control vector. Values are averages (±SD) derived from two independent experiments.

To identify the determinants responsible for the functional differences between HIV-1 group N and SIVcpz Vpus, we analyzed a set of chimeras containing portions of the group N strain YBF30 and that of its closest SIVcpz counterpart, EK505 (Figure 3A) [26]. The YBF30 Vpu was selected for these mapping studies because it is more active in antagonizing tetherin than most remaining N-Vpus. We found that many proteins containing the membrane-proximal cytoplasmic part of the N-YBF30 Vpu were hardly detectable by Western blot (Figure S2). Specifically, the replacement of five amino acid residues in the YBF30 Vpu by the corresponding region of the EK505 Vpu (EYKKI to IYREV, YBF30-IYREV) increased and the reciprocal exchange in the EK505 Vpu (EK505-EYKKI) reduced the detectable levels of Vpu expression (Figure S2). This short region contains two potential lysine ubiquitinylation sites as well as a putative tyrosine-based sorting motif and may affect both Vpu stability and localization. Importantly, several chimeras that were hardly detectable by Western blot were nevertheless active in some functional assays (summarized in Figure 3A) and readily visible in microscopic analyses. This further supports that the detectable levels in immunoblots may not accurately reflect the cellular levels of Vpu expression. More importantly, we found that the TMD of the YBF30 N-Vpu rendered the SIVcpz EK505 Vpu fully active against the particle release restriction imposed by human tetherin (YBF30-EK505; EK505-YBF30 TMD), while the reciprocal exchange disrupted the same activity in the YBF30 Vpu (Figure 3A, B). Thus, similar to M-Vpus [27]–[30], amino acid substitutions in the TMD were responsible for the gain of anti-human tetherin activity by N-Vpus. Since M-Vpus have been shown to interact directly with tetherin in cellular membranes [31]–[33], we examined whether this was also true for N-Vpus. We thus fused the N-terminus (KGN) of Kusabira green to the various Vpus and its C-terminus (KGC) to human tetherin, and performed bimolecular fluorescence complementation (BiFC) assays (Figure 3C, 3D). In this assay, a fluorescent complex is obtained only when the two fragments of Kusabira green are brought into close proximity through direct protein interaction [33]. The results showed that the Vpus of YBF30 and the group M reference strain NL4-3, but not the Vpus of SIVcpz EK505 and MB897, interacted with tetherin in living cells (Figure 3C, 3D).

Figure 3 shows that the TMD of N-Vpus acquired the ability to interact with human tetherin. However, potent tetherin antagonism also requires the proper localization of Vpu [27]–[29]. Thus, we performed confocal microscopy analyses to examine whether the different potencies of M- and N-Vpus in antagonizing tetherin are associated with differences in their subcellular localization. We found that cells expressing the NL4-3 and YBF30 Vpus but not the EK505 Vpu showed greatly reduced levels of tetherin expression at their surface (Figures S3 and S4). Predictably, the TMD of the YBF30 Vpu allowed the EK505 Vpu to keep tetherin away from the cell surface (Figures S3 and S4, bottom panels). Closer examination showed that the M NL4-3, N YBF30 and CPZ EK505 Vpus colocalize to a similar degree with the trans-Golgi network (TGN) marker TGN46 (Figure S5A, S5B). Remarkably, the SIVcpz EK505 Vpu colocalized significantly stronger with tetherin than the YBF30 Vpu and the NL4-3 and EK505 YBF-TMD Vpus showed an intermediate phenotype (Figure S5A, S5C). At first view, it may seem striking that the SIVcpz Vpu that does not interact with tetherin shows the strongest colocalization with the restriction factor. A plausible explanation for this is that (in contrast to the YBF30 Vpu) the EK505 Vpu contains all cytoplasmic domains that are required to target it to the same compartment as tetherin. Obviously, this is also the case for the group M NL4-3 and the fusion EK505 YBF-TMD Vpus but both are capable of degrading tetherin, which may reduce the signal intensities. Altogether, these results are in agreement with the possibility that aberrant localization of N-Vpus contributes to their poor anti-tetherin activity.

Tetherin interaction is mediated by the TMD of Vpu, but subcellular localization and full anti-tetherin antagonism are also dependent on specific residues and motifs in the cytoplasmic domain [27]–[29]. Comparing different chimeras, we found that Vpus containing the TMD of YBF30 but the cytosolic region of SIVcpz EK505 (i.e. YBF-EK505 and EK505-YBF-TMD) were as active in antagonizing tetherin as M-Vpus (Figure 3B, 3E) and also down-modulated CD4, CD1d and NTB-A (Figure 3F to 3H, and data not shown). Moreover, replacement of eight amino acid (aa) residues in the first α-helix of the YBF30 Vpu by those of EK505 (LQEKIKHI by VEEKISQL; YBF30-EK505-H1) was sufficient to partially restore these functions (Figure 3E to H). The analysis of additional fusions between the TMDs of the SJGddd or CK1.62 N-Vpus and the cytoplasmic part of the SIVcpz LB7 or EK505 Vpus confirmed that the chimeric Vpus are substantially more potent in antagonizing human tetherin than each of the parental Vpus (Figure S6). Thus, N-Vpus contain a fully functional TMD that interacts with human tetherin, but lack critical sequences in the cytoplasmic domain that are preserved in group M and at least some SIVcpz Vpus.

### Four amino acid residues in the TMD are critical for anti-tetherin activity of N-Vpus

**Figure 4:**
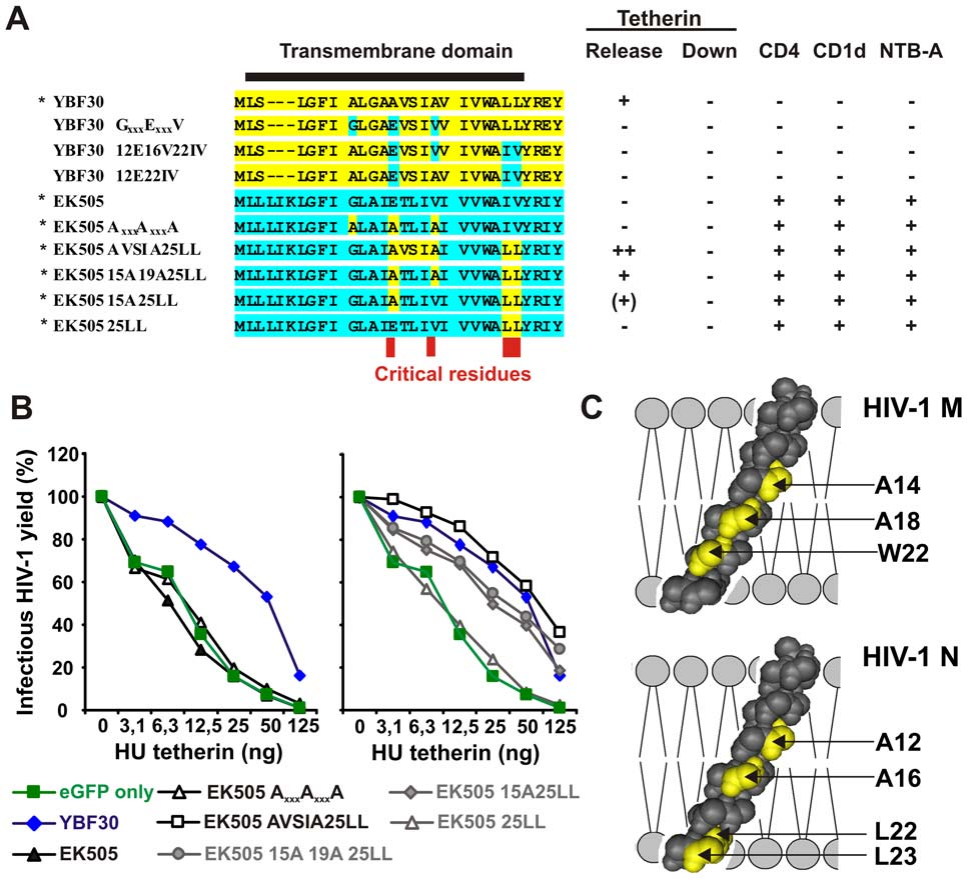
Four residues in the TMD of N-Vpus are critical for anti-tetherin activity. (**A**) The left panel shows an alignment of the TMD of the wild-type and mutant YBF30 (yellow) or EK505 (green) Vpus analyzed and the right panel summarizes their functional activity. Abbreviations: −, no; (+) low; +, modest and ++ high activity. The asterisks indicate the Vpus shown in panel B. (**B**) Infectious virus yield from 293T cells cotransfected with the HIV-1 NL4-3 ΔVpu construct and vectors expressing the indicated *vpu* alleles in combination with various quantities of plasmids expressing tetherin. Shown are average values derived from triplicate infections of TZM-bl indicator cells relative to those obtained in the absence of tetherin (100%). All results were verified by measuring the cell-free and cell-associated p24 levels by ELISA. (**C**) Structure of the Vpu TMD. Residues critical for tetherin antagonism in HIV-1 M and N Vpus are indicated (yellow). Numbering is according to NL4-3 and YBF30 Vpus, respectively.

**Figure 5:**
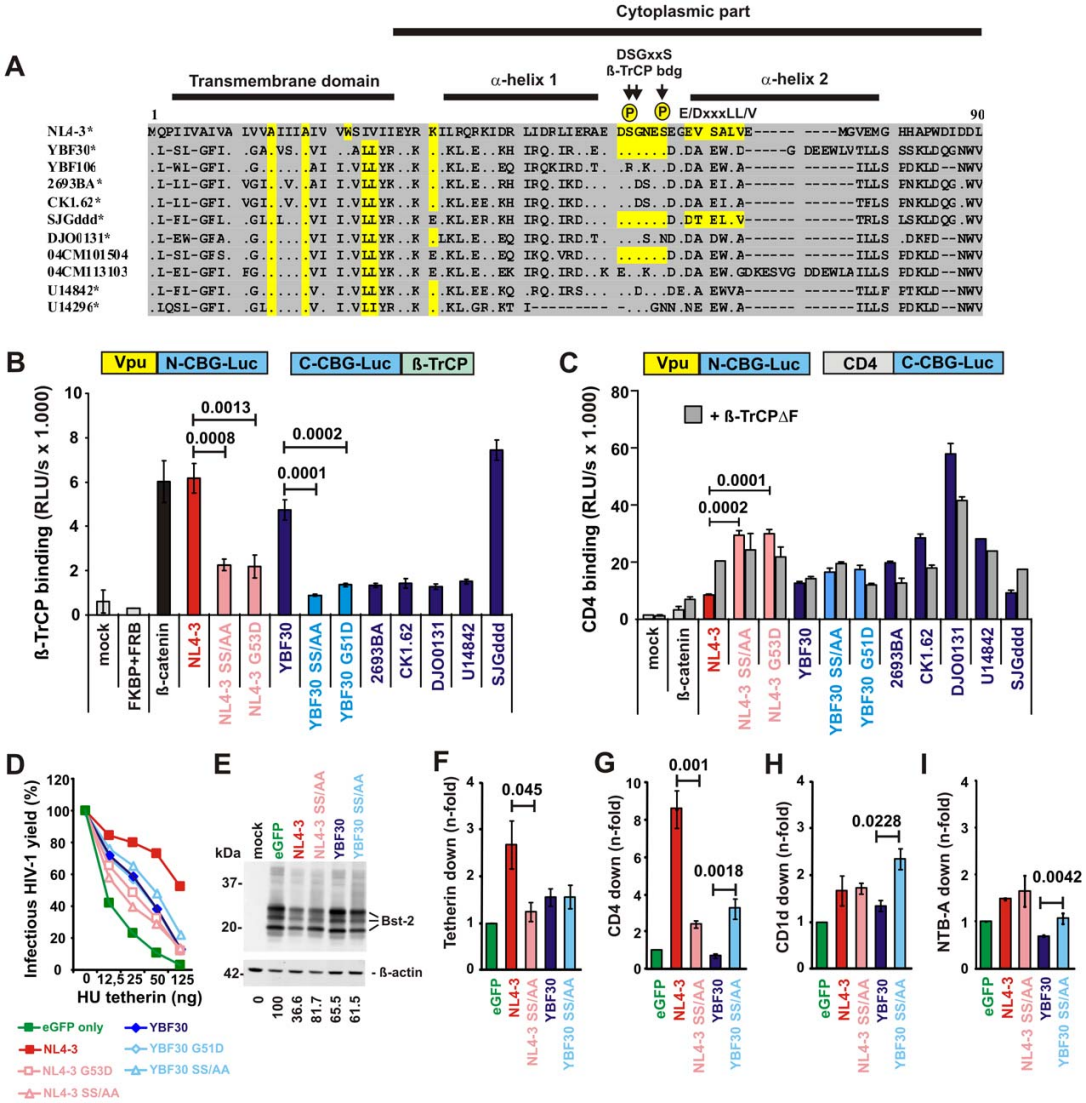
Most N-Vpus lack a functional ß-TrCP binding site. (**A**) Alignment of N-Vpu sequences. The NL4-3 Vpu amino acid sequence is shown on top for comparison. The AxxxAxxxW and AxxxAxxxxxLL residues that are important for anti-tetherin activity of M- and N-Vpus, respectively, a lysine residue that may be required for efficient tetherin antagonism [48], the DSGxxS ß-TrCP interaction site and an E/DxxxLL/I/V/M motif involved in targeting of tetherin for endosomal degradation [42] are indicated in yellow. Dots specify amino acid identity and dashes represent gaps introduced to improve the alignment. (**B**) Interaction of Vpu with ß-TrCP. 293T cells were transfected with equal amounts of plasmids expressing ß-TrCP N-terminally fused to the C-terminal fragment of click beetle green and Vpu C-terminally fused to the N-terminal fragment of click beetle green. ß-catenin served as positive control. After 36 h, click beetle Luciferase activity was determined in living cells by addition of D-Luciferin and quantification of bioluminescence. (**C**) Interaction of Vpu with CD4. 293T cells were transfected with plasmids expressing CD4 C-terminally fused to the C-terminal fragment of click beetle green and Vpu C-terminally fused to the N-terminal fragment of click beetle green and interaction efficiencies were determined as described in panel C. To exclude a bias baused by Vpu-mediated degradation of CD4, a dominant negative mutant of β-TrCP1 isoform 2 lacking the F-box (amino acids 141–193) was cotransfected in a control experiment. This mutant still binds Vpu but lacks the Fbox domain and thus fails to recruit the E3 ubiquitin ligase complex. (**D**) Effect of mutations in the DSGxxS motif of HIV-1 M and N Vpus on infectious virus release in the presence of tetherin. Curves represent the average infection values (n = 3) relative to those obtained in the absence of tetherin (100%). (**E**) Mutations in the ß-TrCP binding site of the M- but not N-Vpu impair tetherin degradation. 293T cells were cotransfected with plasmids expressing the indicated *vpu* alleles and tetherin. Steady state expression levels of tetherin were determined two days later by Western blot analysis. The numbers provided below give the intensities of the tetherin signals relative to the eGFP control lane and were normalized for the ß-actin signal intensity. The results were confirmed in an independent experiment. (**F to I**) Effect of changes in the ß-TrCP binding site of Vpu on down-modulation of tetherin and various receptors. Shown is the Vpu-dependent reduction of (**F**) tetherin, (**G**) CD4, (**H**) CD1d and (**I**) NTB-A surface expression in 293T cells relative to those measured in cells transfected with the eGFP only control vector. Values represent averages (±SD) derived from two independent experiments.

To map the amino acid changes necessary for anti-tetherin activity in the TMD of N-Vpus, we analyzed eight different YBF30 and EK505 Vpu mutants (Figure 4A). The results revealed that four TMD amino acid substitutions (E15A, V19A, I25L and V26L) were sufficient to render the SIVcpz Vpu active against human tetherin, while the reciprocal changes disrupted the effect of the YBF30 Vpu on virus release (Figure 4B). These included two residues in the AxxxAxxxA motif, known to be important for helix-helix interactions [30], as well as two additional changes (LL) in the membrane-proximal hinge region (Figure 4C). Notably, these residues, which are highly conserved among N-Vpus (Figure 5A), overlap only partly with the A14, A18 and W22 residues previously shown to be important for the anti-tetherin activity of group M Vpus [34]. Thus, distinct adaptive changes in the TMD of SIVcpz Vpus seem to restore their interaction with human tetherin. Interestingly, none of the TMD mutations in the YBF30 and EK505 Vpus affected modulation of CD4, CD1d or NTB-A (Figure 4A). Thus, the changes in N-Vpus that conferred anti-tetherin activity are not responsible for the loss of the other Vpu functions.

### Most N-Vpus lack a functional ß-TrCP binding site

HIV-1 M Vpus contain a highly conserved DSGxxS motif that is phosphorylated at the serine residues by casein kinase II and interacts with β-TrCP to recruit the E3 ubiquitin-ligase SCF (Skp1, Cullin, F-box) complex to CD4 and tetherin to mediate their proteasomal or lysosomal degradation [35], [36]. The phenotype of M-Vpus in which the ß-TrCP interaction site has been disrupted, is highly reminiscent of N-Vpus with respect to their lack of CD4 degradation and impaired anti-tetherin activity [17], [37]–[39]. We thus examined the N-Vpu sequences for the presence of the DSGxxS motif and found it to be absent in seven of ten Cameroonian group N strains (Figure 5A). To examine the capability of N-Vpus to directly interact with ß-TrCP, we fused the N-terminal fragment of the click beetle luciferase to the C-terminus of the various Vpus, and the C-terminal fragment of this luciferase to the N-terminus of ß-TrCP (Figure 5B). Interaction between Vpu and ß-TrCP leads to the assembly of the luciferase fragments and thus detectable reporter activity [40]. We found that only the control NL4-3 M-Vpu as well as two N-Vpus (YBF30 and SJGddd), both of which contained intact DSGxxS motifs, interacted with ß-TrCP (Figure 5B). Mutation of the serine phosphorylation sites (S52A/S56A) as well as a G53D substitution found in several N-Vpus disrupted this interaction of NL4-3 and YBF30 Vpus.

Our results showed that most N-Vpus are unable to bind ß-TrCP to target CD4 for proteasomal degradation. However, even the YBF30 and SJGddd N-Vpus that interacted with ß-TrCP were inactive against CD4. To examine whether this was due to an inability to bind CD4, we tested the latter two N-Vpus using the click beetle complementation assay. We considered the possibility that Vpu may degrade the CD4-luciferase reporter construct and thus yield false negative results. To control for this we performed the interaction assays in the presence and absence of a *trans*-dominant negative mutant of ß-TrCP1 (TrCP1ΔFbox) that still binds Vpu but lacks the Fbox domain and thus fails to recruit the E3 ubiquitin ligase complex for proteasomal degradation of CD4 [41]. As shown in Figure 5C, most N-Vpus resulted in increased (albeit variable) levels of luciferase activity in the click beetle complementation assay and thus seemed to interact directly with CD4. Mutations in the ß-TrCP binding site of the NL4-3 Vpu increased signal intensities in the absence but not in the presence of the TrCP1ΔFbox mutant (Figure 5C). This was as expected, since the DSGxxS motif in Vpu is not required for CD4 binding, but critical for its degradation [35]. Such effects were not observed for N-Vpus, suggesting that they are unable to induce ß-TrCP dependent degradation of CD4. Notably, the DJO0131 Vpu, which did not interact with ß-TrCP (Figure 5B) but bound CD4 with the highest efficiency (Figure 5C), was the only N-Vpu that induced a significant albeit modest (2.8-fold) reduction in CD4 cell surface expression. Thus, strong physical association of Vpu with CD4 alone seems sufficient to compromise its transport to the surface, albeit only to a limited degree. The YBF30 and SJGddd Vpus, however, did not down-modulate CD4 cell surface expression, although they were capable of interacting with both ß-TrCP and (weakly) CD4 (Figure 5B, 5C). We therefore examined the contribution of an intact ß-TrCP binding site to various N- and M-Vpu functions. As expected [31], [37], [41], [42], mutations in the DSGxxS motif disrupted CD4 degradation and impaired tetherin antagonism by the NL4-3 M-Vpu (Figure 5D to 5G), while these same changes had no effect on modulation of CD1d and NTB-A (Figure 5H, 5I). In contrast, mutation of the serine phosphorylation sites in the YBF30 N-Vpu had no significant effect on tetherin antagonism and even slightly enhanced CD4, CD1d and NTB-A modulation (Figure 5D to 5I). Co-expression of the *trans*-dominant TrCP1ΔFbox mutant confirmed that the lack of ß-TrCP function reduced the capability of M-Vpus, but not N-Vpus, to counteract tetherin and had generally no effect on CD1d down-modulation (Figure S7). Thus, even those N-Vpus that contain an intact ß-TrCP binding site are functionally impaired because of additional yet-to-be-identified disruptive mutations in their cytoplasmic domain.

### The Vpu of a recently discovered HIV-1 N strain from Togo is a potent tetherin antagonist

**Figure 6:**
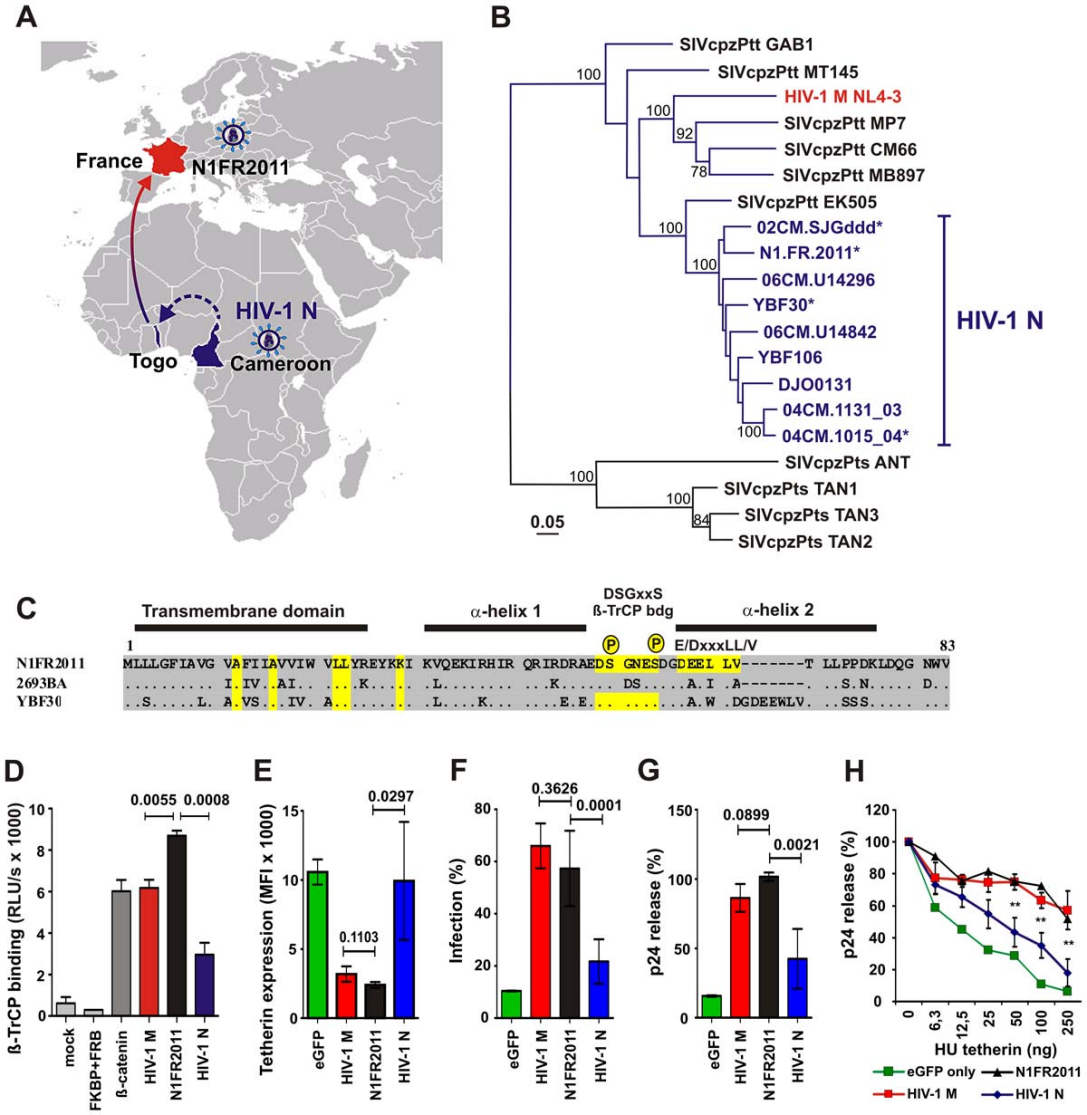
The Vpu of an HIV-1 N strain from Togo is an effective tetherin antagonist. (**A**) Schematic representation of the origin of N1FR2011. This group N infection was diagnosed in Paris in a 57-year old male, 8 days after returning from Togo [3]. All other HIV-1 N infections were identified in individuals from Cameroon. (**B**) Evolutionary relationships among HIV-1 and SIVcpz Env amino acid sequences. Group N viruses are highlighted in blue; those containing an intact DSGxxS motif in their Vpu protein are labelled with an asterisk. The tree was inferred using maximum likelihood methods [58] using a JTT+I+G+F model [59] chosen using ProtTest [60]. Numbers on branches indicate bootstrap support (only values above 70% are shown). The scale bar indicates 0.05 substitutions per site. (**C**) Comparison of the N1FR2011, 2693BA and YBF30 and N-Vpu protein sequences. As indicated, the N1FR2011 Vpu contains intact AxxxAxxxxxLL, DSGxxS and E/DxxxLL/I/V/M sites and a membrane proximal lysine residue (yellow). Dots specify amino acid identity and dashes gaps introduced to improve the alignment. (**D**) Efficient interaction of the N1FR2011 Vpu with ß-TrCP. 293T cells were transfected with plasmids expressing Vpu-N-Luc and C-Luc-ß-TrCP fusions and luciferase activity determined as described in the legend of Figure 4. Shown is the comparison of the luciferase activity obtained for the N1FR2011 Vpu in comparison to group M (n = 3) and N (n = 7) Vpus. Each allele was measured in triplicate and shown are averages ±SDs. (**E**) Efficiency of downmodulation of tetherin in the presence of constructs expressing the indicated Vpus relative to those measured in cells transfected with the eGFP control vector. Values are averages (±SD) derived from three experiments. (**F to H**) Comparison of the effect of N1FR2011 and group N (n = 6) or M (n = 3) Vpu proteins on (**F**) infectious virus production or (**G, H**) viral p24 antigen release determined by (**G**) Western blot or (**H**) ELISA. Values represent the average infection levels or quantities of p24 antigen (n = 3) obtained in the presence of tetherin relative to those obtained in the absence of the restriction factor (100%). Percentages of cell-free p24 were normalized to the levels of cell-associated p24 antigen.

In January 2011, the first HIV-1 group N infection outside Cameroon was diagnosed in a 57-year-old male living in Paris, who presented with fever, rash, lymphadenopathy and genital ulceration about a week after returning from Togo [3]. The virus, termed N1.FR.2011, seemed to have been acquired by heterosexual contact in this West African country (Figure 6A). A month later, his CD4+ T cell count was 219 per µL and the plasma viral RNA load was 4.4 Log10 per mL [3]. Primary HIV-1 N infection was confirmed by full-length viral genome sequencing [3] and bootstrap analysis revealed thatof the N1.FR.2011 is not the result of a recombination with HIV-1 group M (Figure S8). However, the newly identified N1FR2011 strain was not particularly closely related to any other known group N virus (Figure 6B). Inspection of the N1FR2011 Vpu sequence identified an intact DSGxxS motif (Figure 6C) and functional analyses confirmed efficient interaction with ß-TrCP (Figure 6D). In contrast to other N-Vpus, the N1FR2011 Vpu also contains a putative E/DxxxLL/I/V/M trafficking motif in the second α-helix [43] and a lysine residue in the membrane-proximal hinge region [30] reported to be critical for effective Vpu-mediated anti-tetherin activity (Figure 6C). Functional analysis showed that the N1FR2011 Vpu was highly effective in reducing cell surface expression of tetherin (Figure 6E) and in promoting virus release (Figures 6F–H, S9).

**Figure 7:**
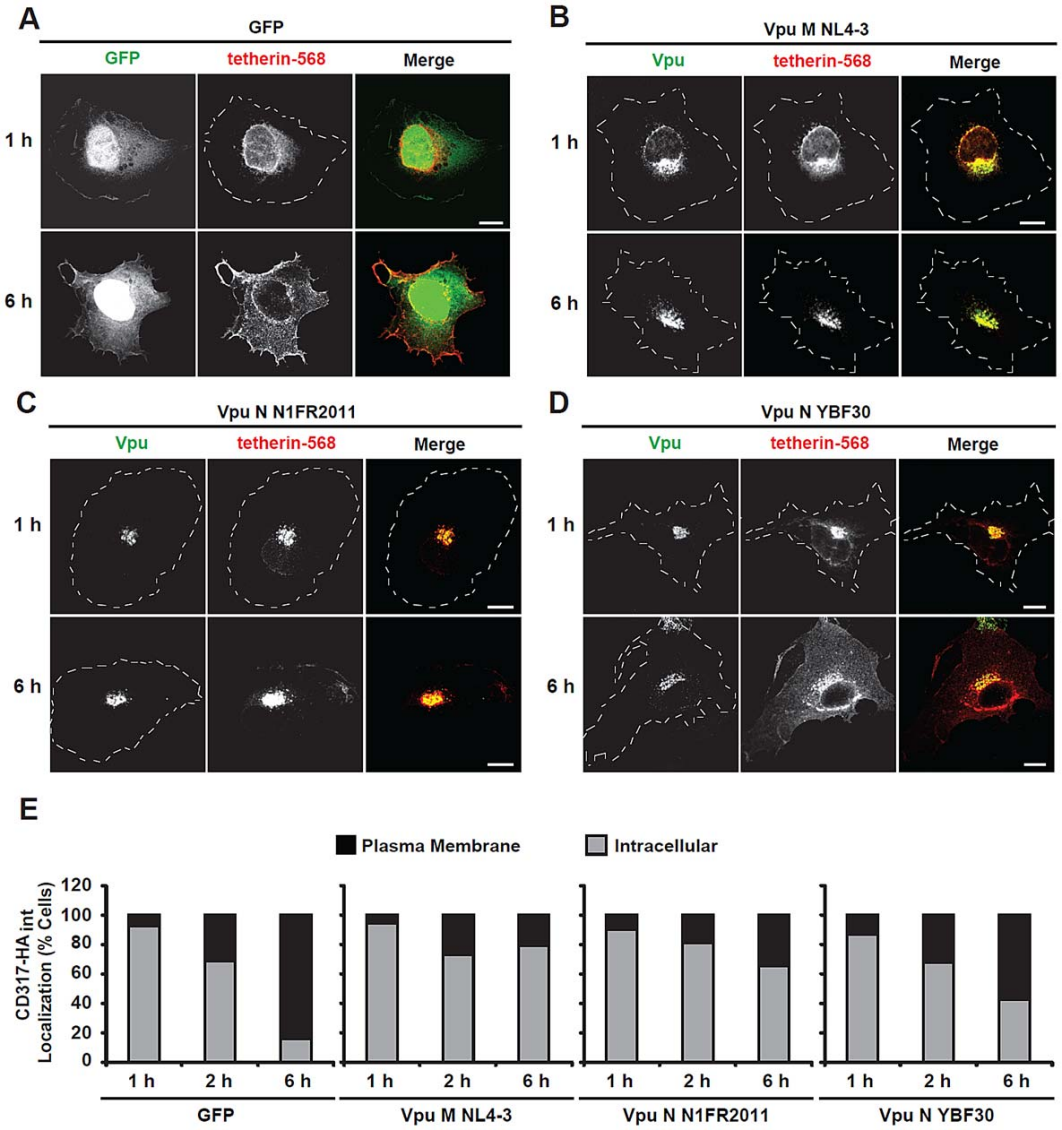
The N1FR2011N-Vpu blocks anterograde transport of tetherin. (**A–D**) The nuclei of HT1080 cells, grown on coverslips, were co-microinjected with an expression plasmid encoding human tetherin with an internal HA tag together with vectors encoding either GFP (**A**) or C-terminally AU1 tagged Vpus: NL4-3 (**B**), N1FR2011 (**C**) and YBF30 (**D**). ∼200 cells were microinjected per plasmid combination. Subsequently, cells were cultivated for 1, 2 or 6 hours and then fixed, permeabilized and stained with an anti-HA mAb followed by an Alexa 568-conjugated secondary a (red staining) to detect newly synthesized tetherin together with an anti-AU1 rab followed by Alexa 488-conjugated secondary antibody (green staining) to detect newly synthesized Vpu proteins. Microphotographs shown are representative for three independent experiments. Cell circumferences are indicated. Scale bars: 10 µm. (**E**) Stainings were categorized into cells, which, besides intracellular staining, also displayed a clear plasma membrane staining (“plasma membrane”) or cells with an exclusively intracellular staining (“intracellular”). Histogram bars depict the relative percentage of cells for each time point from at least 150 cells that were analyzed out of three independent microinjection experiments.

**Figure 8:**
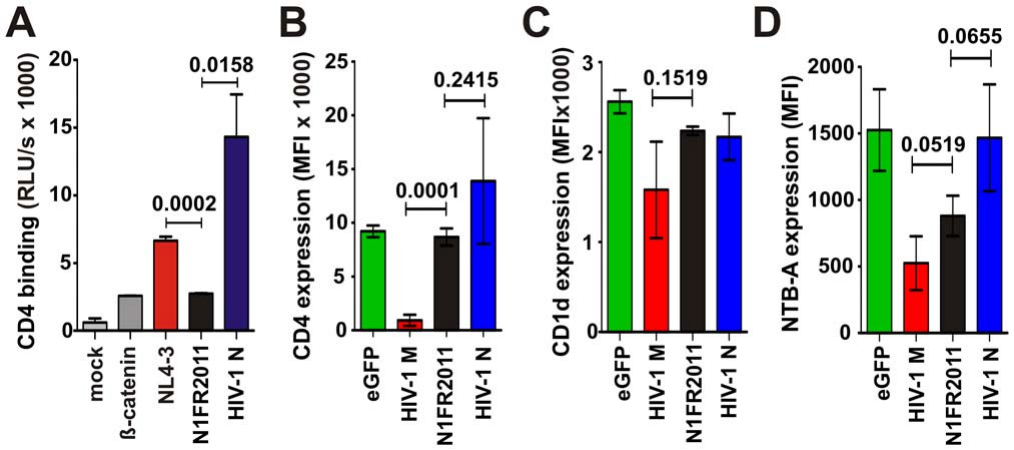
Effect of the N1FR2011N-Vpu on CD4, CD1d and NTB-A. (**A, B**) The N1FR2011 Vpu does not interact with CD4 (**A**) or reduce its cells surface expression (**B**). 293T cells were transfected with plasmids expressing Vpu-N-Luc and CD4-C-Luc and interaction efficiencies were determined as described in the Experimental Procedures. (**C, D**) Vpu-dependent reduction of CD1d (**C**) and NTB-A (**D**) surface expression in 293T cells. Shown are the levels of receptor cell surface expression for group M (n = 3), N1FR2011, and Cameroonian N (n = 7) Vpus relative to those measured in cells transfected with the eGFP only control vector. Each allele was measured in triplicate and shown are averages ±SDs.

To examine by which mechanism the N1FR2011 Vpu antagonizes tetherin, we employed a microinjection approach that allows for the analysis of anterograde transport of *de novo* expressed tetherin by confocal microscopy [44]. In the absence of Vpu, newly synthesized tetherin was efficiently transported to the plasma membrane within 6 hours in 85% of cells analyzed (Figure 7A, 7E). Both the control NL4-3 M-Vpu and the N1FR2011 N-Vpu efficiently trapped tetherin at the TGN and prevented its forward transport to the cell surface (Figure 7B, 7C, 7E; only 20–35% of cells with tetherin at the plasma membrane 6 hours post-injection). The YBF30 N-Vpu also affected tetherin transport to the plasma membrane, albeit with substantially lower efficiency and with less pronounced TGN accumulation of the restriction factor (Figure 7D, 7E, up to 60% of cells with tetherin at the plasma membrane 6 h post injection). Together, these data demonstrated that the N1FR2011 Vpu counteracted tetherin about as efficiently as M-Vpus via disruption of anterograde transport and was significantly more potent in this function than the remaining N-Vpus. Nonetheless, this N-Vpu did not bind CD4 (Figure 8A) and was thus unable to reduce its cell surface expression (Figure 8B). Like Vpus from Cameroonian group N viruses, it had only marginal effects on CD1d cell surface expression (Figure 8C). However, it significantly reduced cell surface expression of NTB-A, albeit with lower efficiency than M-Vpus (Figure 8D). Thus, the Vpu protein of this HIV-1 N strain from Togo is superior to those of Cameroonian group N strains in two functions, i.e. tetherin antagonism and down-modulation of NTB-A.

## Discussion

HIV-1 group N is an example of a (thus far) poorly successful SIVcpz zoonosis, which until recently has only been detected in about a dozen individuals from Cameroon [2]–[4]. In the present study, we analyzed the function of Vpu proteins from these viruses to elucidate to what extent they are impaired in tetherin antagonism and down-modulation of CD4, CD1d and NTB-A. We also examined the Vpu protein of one unusual group N strain that was only recently discovered in a French patient who most likely became infected in Togo [3]. This N strain represents a very recent infection and the only documented transmission case outside of Cameroon. We thus reasoned that this strain may provide insights into whether Vpu, which has been implicated as a determinant of viral transmission fitness [12], [45], continues to be under strong host specific selection. Our data show that N-Vpus have evolved a transmembrane domain that efficiently interacts with human tetherin. However, most also contain disruptive changes in their cytoplasmic region, which are not only responsible for their inability to down-modulate CD4, but also attenuate their anti-tetherin activity. Only the most recently identified HIV-1 N strain from Togo expressed a Vpu that was fully active against tetherin, yet remained inactive against CD4 because it fails to interact with this receptor. These findings indicate that group N viruses are still in the process of adaptation, with strong host selection pressures shaping the function of their Vpu proteins, especially their ability to clear the tetherin barrier.

One surprising finding was that replacement of the TMD domain of the Vpu protein of SIVcpz EK505, which represents the closest known chimpanzee relative of HIV-1 group N (Figure 5B), with that of an N-Vpu yielded a highly active protein with functional properties indistinguishable from M-Vpus (Figure 3). Similar results were obtained with additional chimeras between the TMDs of N-Vpus and the cytoplasmic parts of other SIVcpz Vpus (Figure S6). Thus, the TMD of N-Vpus seems to be fully capable of interacting with human tetherin, while domains critical for this same function are conserved in the cytoplasmic domain of some SIVcpz Vpu proteins. The latter is striking since SIVcpz Vpus are inactive against both human and chimpanzee tetherin [19]. It also came as a surprise that just four amino acid changes in the TMD of the EK505 Vpu (E15A; V19A; IV25/26LL) rendered it active against human tetherin without disrupting the CD4, CD1d and NTB-A down-modulation functions (Figure 4). These data suggest that close SIVcpz relatives of HIV-1 N would require only a few mutations in the TMD to evolve a fully functional Vpu. Yet, this is not what happened during the evolution of HIV-1 group N. While the required TMD mutations are present in all N-Vpus, most of them also contain disruptive changes in their cytoplasmic domain, which render them functionally inferior to group M Vpus. Thus, for reasons that remain elusive, the N-Vpu precursor does not seem to have taken the most direct route towards full functional activity, which has been achieved by the group M Vpu precursor.

Our finding that N-Vpus have evolved functional TMDs, but contain disruptive changes in their cytoplasmic region seems to contradict the principle that only mutations that increase viral fitness should be selected for and thus become fixed. In this context, the changes in the DSGxxS ß-TrCP binding site of N-Vpus seem most striking, because this motif is critical for CD4 degradation not only for M-Vpus but also CPZ-Vpus. Thus, the selective advantage of preserving the CD4 degradation function, which is independent of the host species [19], should have ensured the maintenance of the DSGxxS site. One explanation is that the progenitor of group N viruses encoded a grossly defective Vpu. However, at least for the CD4 degradation function this seems unlikely since the CD4 degradation activity is highly conserved in SIVcpz Vpus [19]. Moreover, the predicted Vpu amino sequence of the most recent common ancestor of group N viruses contains an intact DSGxxS ß-TrCP binding site and reduces the cell surface expression of CD4 (data not shown). Alternatively, these alterations in N-Vpus may have emerged because they increase other yet-to-be-defined N-Vpu functions, such as its ability to modulate innate immune signaling [46], [47], or because the introduced changes alter the Kozak sequence of the Env initiation codon and/or other signatures in the overlapping signal peptide. Whatever the reasons, the unusual functional evolution of N-Vpus suggests that maintaining Vpu-mediated CD4 degradation is less critical than gaining tetherin antagonism, possibly because two other viral gene products (Env and Nef) also reduce CD4 cell surface expression. Finally, the emergence of a group N strain outside of Cameroon that encodes a Vpu with potent anti-tetherin activity suggests that this viral lineage has the potential to evolve towards increased transmission fitness. Whether the Togo strain is an exception or the result of serial adaptations is not known. Notably, in closely related group N viruses infecting a couple [48], the ß-TrCP interaction site was found in the Vpu sequence obtained from the wife but not from the husband (04CM.1015_04 and 04CM.1131_03, Figure 5A). This suggests restoration of the DSGxxS motif following transmission since the husband was most likely infected first and subsequently transmitted the virus to his wife [48].

**Figure 9:**
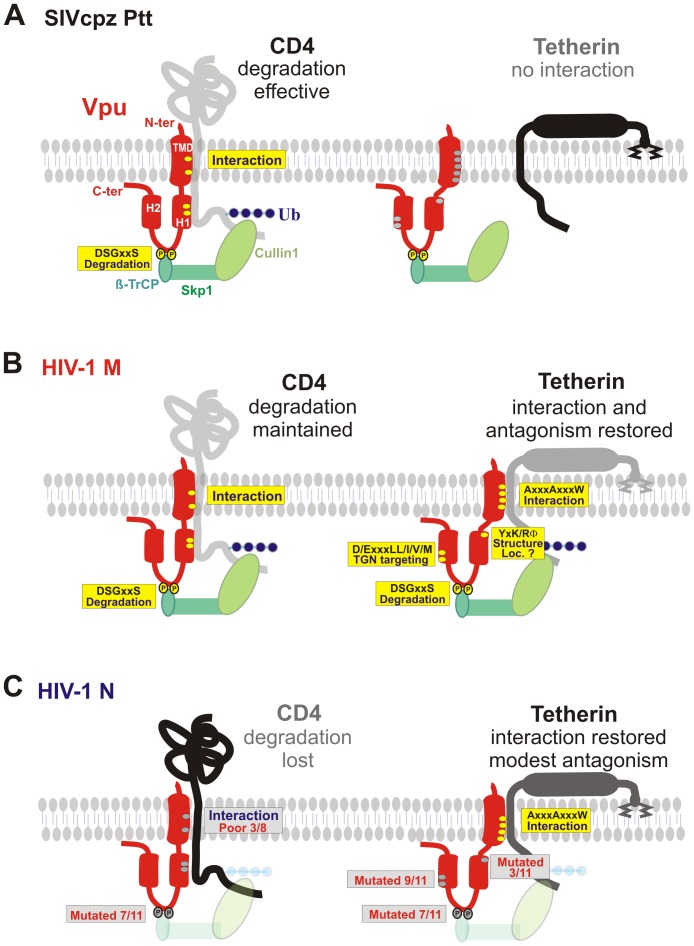
Schematic presentation of SIVcpz and HIV-1 Vpu motifs and interactions. (**A**) SIVcpz Vpus contain a functional DSGxxS ß-TrCP binding site and adapt CD4 to the E3 Skp1/Cullin1 ubiquitin-ligase to induce its degradation. In contrast, the TMD domain of SIVcpz Vpus is unable to intact with tetherin. The dots in the schematic Vpu structure indicate the approximate position of amino acid residues involved in the respective functions (yellow, conserved and functional; grey, variable and/or non-functional). (**B**) During adaptation to humans, M-Vpus acquired several changes in the TMD that allow efficient interaction with the TMD of tetherin and permit its degradation and reduction of cell surface expression. Some amino acids and functional domains in the cytoplasmic region, such as a basic lysine or arginine residue in a putative YxxΦ motif in the membrane proximal hinge region, the DSGxxS ß-TrCP interaction domain and a E/DxxxLL/I/V/M site in the 2nd α-helical region that are involved in TGN targeting and effective antagonism of tetherin are highly conserved in M-Vpus. (**C**) HIV-1 N Vpus also evolved the capability to interact efficiently with HU tetherin after zoonotic transmission and the residues in the TMD involved in this interaction (E15A, V19A and IV25/26LL) are highly conserved (11/11). In contrast, most N-Vpus contain mutations in various cytoplasmic domains, such as the lysine residue (3/11), the DSGxxS motif (7/11) and the E/DxxxLL/I/V/M site in the 2nd α-helical region (9/11). Furthermore, some N-Vpus interact only weakly with CD4. Together, these disruptive mutations in the cytoplasmic region largely explain the poor activity of HIV-1 N Vpus.

The adaptive routes of HIV-1 N Vpu function seem strikingly different from those of HIV-1 groups M, O and P (summarized in Figure 9). SIVcpz and SIVgor Vpus degrade CD4, yet do not antagonize tetherin because these viruses utilize Nef to counteract this restriction factor (Figure 9A). O-Vpus and P-Vpus still have functional properties that are highly similar to those of their SIV counterparts [19], [23]. In contrast, M-Vpus acquired changes in their TMD and possibly elsewhere that “restored” anti-tetherin activity while maintaining the CD4 degradation function [19], [34]. As shown in Figure 9B, full Vpu activity requires a variety of functional domains and interactions. To promote CD4 degradation, Vpu must interact with CD4 via residues located in its transmembrane and α-helix 1 regions and it must also bind to ß-TrCP to target the receptor for proteasomal degradation [35]. To antagonize tetherin, Vpu has to interact with this restriction factor via its TMD and this interaction seems sufficient for modest anti-tetherin activity [16], [28], [31], [37], [42], [43], [49]. Effective tetherin antagonism, however, seems to require several domains in the cytoplasmic domain of Vpu, such as the DSGxxS motif [20], [29], [37], [41], [42], a putative trafficking motif (E/DxxxLL/I/V/M) [43], and possibly a basic residue in a putative YxxΦ motif in the membrane-proximal hinge region of the cytoplasmic tail [29], [49]. All of these residues and interaction sites are highly conserved in M-Vpus but frequently absent in N-Vpus (Figures 9C, S10). A possible explanation is that these cytoplasmic domains have to act in concert to perform their respective role in Vpu function. For example, an intact ß-TrCP binding site may only facilitate Vpu-mediated degradation of CD4 (and/or tetherin) if other functional domains are also present that ensure proper localization of Vpu and efficient interaction with its cellular targets. Thus, disruption of one functional Vpu motif may facilitate the accumulation of mutations in others because these are not associated with further loss of function. This would explain why most N-Vpus harbor mutations in multiple cytoplasmic domains, suggesting that a combination of changes is required to achieve a significant gain of function. Nonetheless, the most recently described HIV-1 N N1FR2011 strain expresses a Vpu protein in which all cytoplasmic domains known to be critical for effective anti-tetherin activity of M-Vpus, such as a basic K residue, as well as DSGxxS and D/ExxxLL/I/V/M motifs are intact (Figure 6C). It thus seems that N-Vpus are partially adapted to human tetherin because direct Vpu-tetherin interaction is sufficient to promote virion release. Nonetheless, the effects are modest because most N-Vpus lack functional domains required for correct subcellular localization and tetherin degradation.

Although HIV-1 N strains have not spread extensively, they can cause CD4+ T cell depletion and AIDS [4], [5], [48]. This is also true for group N viruses whose Vpus lack the DSGxxS motif (Table S1). Thus, effective tetherin antagonism and CD4 degradation do not seem to be required for efficient viral replication and disease progression *in vivo*. In this context it should be noted that M-Vpus that contain mutations in the DSGxxS domain are still capable of promoting HIV-1 replication in CD4+ T cells and human lymphoid tissues but not in macrophages [38]. Thus, modest anti-tetherin activity may be sufficient to promote virus release in primary cells expressing relatively low levels of this restriction factor. This may explain why the residues in the TMD of N-Vpus involved in tetherin antagonism are highly conserved (Figure 5A) and why intact full-length *vpu* genes are found in most HIV-1 N strains although they are usually only marginally active in *in vitro* assays for Vpu function. Interestingly, some SIVcpz Vpus, including those of strains most closely related to HIV-1 groups M and N, are capable of down-modulating CD1d and/or NTB-A, whereas most N-Vpus lack these activities. The effects in transiently transfected 293T cells were highly reproducible but also rather modest; thus, these recently identified Vpu activities clearly need to be examined in virally infected primary cells. Increased susceptibility to NK cells and/or NKT cells may represent yet another reason for the poor spread of group N viruses, particularly since increased NK cell activity has been correlated with protection from HIV-1 infection [50]. Thus, further studies of the effects of M- and N-Vpus on innate immune mechanisms in primary target cells of HIV-1 seem warranted.

In summary, we show that N-Vpus interact with tetherin and can (at least to some extent) promote virus release, indicating partial adaptation to humans. However, the gain of function mutations in the TMD occurred concomitantly with the disruption of functional motifs in their cytoplasmic domain. The reasons for this are unclear and may be inherent in the genetic background of the SIVcpz strain that gave rise to group N and/or selection pressure in overlapping Env regions. The number and nature of the disruptive changes in the cytoplasmic domain of N-Vpu vary, suggesting ongoing diversification, which may only be successful in increasing Vpu function in exceptional cases. The finding of a group N variant from Togo that is fully active against tetherin, yet still lacks the CD4 down-modulation function, is consistent with this hypothesis. We have previously speculated that potent Vpu-mediated tetherin antagonism may be more relevant for HIV-1 transmission than for viral pathogenesis, because effective virion release may reduce the shedding of infectious virions into genital fluids [12]. It will thus be of great interest to monitor whether additional group N strains with fully restored anti-tetherin activity will emerge.

## Materials and Methods

### Ethical statement

Studies involving human material were reviewed and approved by the University of Ulm Institutional Review Board, and individuals and/or their legal guardians provided written informed consent prior to donating blood.

### Expression vectors

Bi-cistronic CMV-based pCGCG expression vectors coexpressing Vpu or tetherin and the enhanced green fluorescent protein (eGFP) and DsRed2, respectively have been described previously [19]. The coding regions of SJGddd, U14842 and U14296 *vpu* were synthesized from published sequences. DJO0131 *vpu* was amplified from a proviral clone. Splice-overlap-extension or standard PCR with primers introducing *XbaI* and *MluI* restriction sites flanking the reading frames was used to generate mutant or chimeric *vpu* or CD1d and NTB-A coding PCR fragments for cloning into the pCGCG vector. To inhibit β-TrCP mediated recruiting of the E3 ubiquitin-ligase to Vpu, a dominant negative mutant of β-TrCP1 isoform 2 lacking the F-box (amino acids 141–193) was synthesized by Genscript (Genscript, Piscataway, USA) and cloned into the pCGCG vector coexpressing DsRed2 via an IRES. All PCR-derived inserts were sequenced to confirm that no undesired nucleotide changes were present.

### Proviral constructs

Proviral HIV-1 NL4-3 vectors expressing various *vpu* alleles independently of overlapping *env* gene sequences were as described previously [19]. The 02CM-DJO0131 strain, reported in Bodelle *et al.* [2] was selected for the construction of the first HIV-1 group N molecular clone. The near full-length sequence of this strain (Genbank accession number AY532635) was originally generated by direct sequencing of overlapping amplification products obtained by RT-PCR from plasma vRNA, thus is likely to represent the consensus of actively replicating quasispecies. The published sequence contains only 5 ambiguous bases, including 2 synonymous in the pol and nef genes, respectively, and 3 non-synonymous in the gag gene. To generate the full-length molecular clone sequence, the most common nucleotide in the HIV-1 group N consensus was selected for the base positions with synonymous ambiguities and the most common amino acid in the HIV-1 group N consensus was selected for the base positions with non-synonymous ambiguities. The resulting reconstructed provirus containing complete 5′ and 3′ LTR (9720 base pairs) was chemically synthesized in three overlapping fragments (purchased from Blue Heron Biotechnology, Bothell, WA); the full-length clone was assembled using the unique BstZ17I (nucleotide position 2966) and PshAI (nucleotide position 8449) restriction enzyme sites, and inserted between the MluI and NotI restriction enzyme sites in the pCR-XL vector. The resulting full-length proviral clone was transfected into 293T cells to generate cell-free virus stocks and the TZM-BL-based single-round infectivity assay was used to determine the infectious titer of the DJO0131 virus stock. The replication competence of the transfection-derived DJO0131virus was tested in primary human CD4+ T-cells cultures. DJO0131 virus replicated to high titers, with replication kinetics similar to those of HIV-1 SG3 and YU2 laboratory controls. A *vpu*-deficient variant was generated by introducing a stop codon at position 2 of the *vpu* reading frame. The start codon was not mutated to avoid alteration of *env* expression. To generate a *nef*-deficient mutant, the start codon of *nef* and codon 20 coding for an in frame Met were changed to TAG. A frameshift (+1) disrupting an EcoRV site in *env* was introduced to generate an *env*-deficient variant of HIV-1 N DJO0131.

### Cell culture

293T cells were maintained in Dulbecco's modified Eagle medium (DMEM) supplemented with 10% heat-inactivated fetal bovine serum, 350 µg/ml L-glutamine, 120 µg/ml streptomycin sulfate and 120 µg/ml penicillin. 293T cells were transfected by the calcium phosphate method as described previously [51] or Lipofectamine LTX reagent. TZM-bl cells were kindly provided by Drs. Kappes and Wu and Tranzyme Inc. through the NIH AIDS Reagent Program and were kept in DMEM supplemented with 10% heat-inactivated fetal bovine serum, 350 µg/ml L-glutamine, 120 µg/ml streptomycin sulfate and 120 µg/ml penicillin. TZM-bl cells express large amounts of CD4, CCR5 and CXCR4 and contain the ß-galactosidase gene under the control of the HIV-1 promoter [52]–[54]. Human peripheral blood mononuclear cells (PBMC) from healthy human donors were isolated using lymphocyte separation medium (Biocoll Separating Solution, Biochrom), stimulated for 3 days with PHA (2 µg/ml) and cultured in RPMI1640 medium with 10% FCS and 10 ng/ml IL-2 prior to infection.

### Flow cytometric analysis

To determine the effect of Vpu on CD4, CD1d, NTB-A and tetherin cell surface expression, 293T cells were transfected by the calcium phosphate method in triplicate with 1 µg of a CD4, CD1d, NTB-A, or tetherin expression vector and 5 µg of pCGCG eGFP/Vpu constructs expressing eGFP alone or together with Vpu. Two days post-transfection CD4, CD1d, NTB-A or tetherin expression was examined by FACS analysis, essentially as described previously [19], [55]. An allophycocyanin-conjugated anti-human CD4 antibody (Invitrogen; MHCD0405), a phycoerythrin-conjugated anti-CD1d antibody (BD 550255) or an APC-conjugated anti-SLAM6 antibody (R&D FAB19081A) was used for staining. For staining of tetherin an unconjugated anti-HM1.24 antibody (Chugai Pharmaceuticals) and an APC-conjugated secondary anti-mouse antibody (Invitrogen, A865) were used. Fluorescence of stained cells was detected by two-color flow cytometry and Vpu-mediated CD4, CD1d, NTB-A or tetherin down-modulation was calculated as described previously for the functional analysis of *nef* alleles [55]. Briefly, the mean fluorescence intensities were determined for cells showing specific ranges of eGFP expression. The fluorescence values obtained for cells transfected with the control construct expressing only eGFP was compared with the corresponding number obtained for cells co-expressing Vpu and eGFP to determine the efficiency of CD4, CD1d, NTB-A or tetherin degradation. The same eGFP gating was used in all calculations. To determine the effect of Vpu on tetherin surface expression levels in primary cells, PHA-stimulated PBMCs were transduced with VSVg-pseudotyped HIV-1 M NL4-3 IRES *env* constructs containing different *vpu* genes (19) or HIV-1 N DJO0131 and mutants thereof. Three days after transduction, PBMCs were stimulated with PHA again and cultured for another 24 h. After staining of surface tetherin, cells were permeabilized and intracellular p24 was stained with a PE-conjugated antibody (Beckman coulter). Down-modulation of tetherin was calculated as described above.

### Tetherin antagonism

To determine the capability of Vpu to antagonize tetherin, 293T cells were seeded in six-well plates and transfected with 2 µg of NL4-3 ΔVpu IRES eGFP, 500 ng Vpu expression plasmid and different dilutions of tetherin expression vectors (125, 50, 25, 12.5, 6.25 and 3.125 ng). A pCGCG vector expressing DsRed2 only was used to equalize the DNA concentrations. At two days post-transfection supernatants were harvested and the yield of infectious HIV-1 was determined by a 96-well infection assay on TZM-bl indicator cells, a p24 ELISA or a p24 Western Blot as described previously [19], [51].

### Western blot

To monitor Vpu expression, 293T cells were transfected with 5 µg of vector DNA co-expressing eGFP and AU-1 tagged Vpus. Two days post-transfection cells were harvested, lysed in RIPA (1% NP-40, 0.5% Na-DOC, 1% SDS, 0.15 M NaCl; 50 mM Tris-HCl pH 7.4; 5 mM EDTA) and cell lysates were separated in 4–12% Bis-Tris gels (Invitrogen). After gel electrophoresis, proteins were transferred onto PVDF membranes and probed with AU-1 antibody (Covance, MMS-130P). Subsequently, blots were probed with anti-mouse or anti-rabbit IRDye Odyssey antibodies (926-32210, 926-32221) and proteins detected using a LI-COR Odyssey scanner. For internal controls, blots were incubated with antibodies specific for eGFP (290-50, Abcam) and β-actin (8227-50, Abcam). To examine the levels of tetherin expression, 293T cells were transfected with 5 µg of N-terminally AU1-tagged tetherin plasmids coexpressing DsRed2. At 48 hours post-transfection, cells were lysed with modified RIPA buffer (150 mM NaCl, 50 mM Tris-HCl, 1% NP-40, 0,5% Na-Desoxycholate, 1% SDS, 5 mM EDTA, protease inhibitor cocktail). Lysates were boiled at 95°C for 10 min, separated on 4–12% Bis-Tris gradient acrylamide gels, blotted onto PVDF membranes and probed with anti-BST-2 (Chugai Pharmaceutical Co.), anti-RFP (62341-100, abcam) or anti-ß-actin (8227-50, Abcam). Subsequently, blots were probed with anti-mouse or anti-rabbit IRDye Odyssey antibodies (926-32210, 926-32221) and proteins detected using a LI-COR Odyssey scanner.

### Microscopy

HeLa cells were transfected with different AU1-tagged *vpu* alleles and a TGN- marker plasmid (pAcGFP1-Golgi, Clontech), respectively. Cells were stained 48 h after transfection with Lipofectamine LTX Reagent or calcium phosphate. Briefly, cells were fixed with 4% PFA and permeabilized with 0.5% Saponin. After blocking with BSA and glycine, Vpu and tetherin were stained with an anti-AU1 (Covance) and anti-tetherin (Chugai) antibody, respectively. Secondary antibodies conjugated to Alexa Fluor 647 and Alexa Fluor 568 were used for detection. A confocal microscope (LSM 710, Zeiss) with the corresponding software (LSM 710 Release version 5.5SP1, Zeiss) was used for analysis.

### Bimolecular Fluorescence Complementation assay (BiFC)

To detect interaction of Vpu with tetherin, we used the BiFC technique [33]. Briefly, two fragments of Kusabira-Green (KG) fluorescent protein are brought together by the interaction of two proteins fused to these fragments, thus allowing specific detection of interaction in living cells (Amalgaam). Vpus were cloned into phmKGN-MN and tetherin into phmKGC-MC via BamHI/NotI. 293T cells were cotransfected with 2.5 µg of the vpu construct and 2.5 µg of the tetherin construct. Two days post transfection, cells were analysed by flow cytometry for their KG expression. To exlude a bias by Vpu-mediated degradation of tetherin, cells were stained for tetherin and the mean fluorescence intensity (MFI) of KG was normalized to the MFI of tetherin. For microscopic observation, cells were fixed with 4% PFA for 30 min. and images were acquired with confocal microscope (LSM 710, Zeiss).

### Click beetle assay

The click beetle luciferase heteroprotein fragment complementation assay allows the real-time analysis of protein-protein interactions in living cells [40]. pCBG-C_ß-TrCP1 and pß-catenin_CBG-N constructs encoding the N- or C-terminal fragment of click beetle green (CBG) were kindly provided by Piwnica-Worms. *SalI* and *BamHI* restriction sites were added to the *vpu* alleles by PCR and standard cloning techniques were used to insert the *vpu* alleles into the pCBG-C vector replacing the ß-catenin gene. Splicing by overlap extension PCR (SOE-PCR) was used to fuse the C-terminal CBG-fragment to the C-terminus of human CD4. The ß-TrCP1-CBG-C gene was replaced by the CD4-CBG-C fusion gene via *HindIII* and *XbaI* digestion. The Luciferase assay was essentially performed as described before [40]. Briefly, 293T cells in white 96-well plates with clear bottom were transfected with equal amounts of the click beetle green constructs. 36 hours after transfection the cells were washed once and then incubated in MEBSS buffer (Modified Earle's balanced salt solution: 144 mM NaCl, 5.4 mM KCl, 0.8 mM MgSO_4_, 0.8 mM NaH_2_PO_4_, 1.2 mM CaCl_2_, glucose 5.6 mM, and HEPES 4 mM [pH 7.4]) containing 1% heat-inactivated fetal bovine serum and 150 mg/ml D-Luciferin. Photon flux was quantified at room temperature with a SAFAS Xenius spectrofluorimeter for 1 min.

### Sequence analysis

Vpu amino acid sequences were aligned using multiple sequence alignment with hierarchical clustering (http://multalin.toulouse.inra.fr/multalin/). WebLogo 2.8.2 (http://weblogo.berkeley.edu) was used to create frequency plots. Vpu sequences were obtained from the HIV Sequence Database (www.hiv.lanl.gov).

### Recombination analysis

Available full-length HIV-1 group N sequences were aligned with SIVcpz*Ptt* EK505 as well as HIV-1 group M subtype reference sequences (www.hiv.lanl.gov/content/sequence/NEWALIGN/align.html) using CLUSTAL W version 2.1 [56]. Regions of ambiguous alignment were removed and the resulting alignment was analyzed for evidence of recombination between the HIV-1 N N1FR2011 strain and HIV-1 group M subtype reference sequences using SIMPLOT version 3.5.1 [57].

### Tetherin anterograde biosynthetic transport assay

HT1080 cells grown on coverslips, were microinjected into their nuclei with an AIS 2 microinjection apparatus using pulled borosilicate glass capillaries in principle as reported [44]. Plasmids encoding an tetherin with an internal HA-tag at position 154 in the extracellular domain (CD317-HA_int_) and either GFP or C-terminally AU-1 tagged Vpu's were mixed in water at concentrations of 7 ng/µl and 10 ng/µl, respectively, and co-injected. Following microinjection, cells were cultured for 1, 2 or 6 hours at 37°C to allow protein expression and trafficking. At the indicated time points cells were fixed with 4% paraformaldehyde/PBS and CD317-HA_int_ molecules were detected using a mouse anti-HA mAb (Santa-Cruz) followed by a goat anti-mouse Alexa568 secondary antibody (Invitrogen). Newly synthesized Vpu molecules fused to AU-1 were detected using rabbit anti-AU1 (Covance) and anti-rabbit Alexa 488 secondary antibody (Invitrogen). Stained cells were imaged with a Zeiss LSM 510 confocal microscope.

### Statistical analysis

All statistical calculations were performed with a two-tailed unpaired or paired Students-t-test using Graph Pad Prism Version 5.0. P values<0.05 were considered significant. Correlations were calculated with the linear regression module.

## Supporting Information

Figure S1 **Vpu expression and correlation between various Vpu activities.** (**A**) Expression of selected HIV-1 and SIV Vpu proteins. 293T cells were transfected with expression plasmids encoding the indicated AU1-tagged Vpus and eGFP. Mock transfected cells were used as negative controls; ß-Actin and eGFP expression levels were analyzed to control for loading and transfection efficiency, respectively. (**B**) Correlation between various Vpu activities. The upper panel shows results obtained for the seven N-Vpus and the lower panel shows data obtained for all HIV-1 and SIVcpz Vpus analyzed. (**C**) Vpu expression levels detected by immunoblot do not correlate with functional activity. Vpu expression levels were quantified from the Western blots shown in panel A and normalized to ß-actin. N-fold down specifies the reduction of mean fluorescence intensities obtained for the different markers in the presence of Vpu compared to those measured in cells transfected with the eGFP control construct. Viral release in the presence of tetherin is expressed as the percentage of viral infectivity in the absence of the restriction factor (100%).
(TIF)

Figure S2 **Expression of Vpu chimeras and mutants.** Expression of the indicated wild-type, mutant and chimeric Vpu proteins was determined as described in the legend to Fig. S1. The amino acid sequences of the mutant Vpus are shown in Figs. 3A and 4A. Similar results were obtained in an independent experiment.
(TIF)

Figure S3 **Overview on the cellular localization of tetherin and various Vpu proteins.** Confocal immunofluorescence images of HeLa cells transfected with constructs expressing the indicated Vpu variants. Two days post-transfection, cells were fixed and permeabilized for intracellular staining of tetherin (red), Vpu (blue) and the TGN (green). Images show an overview on confocal acquisitions. The squares in the right panels indicate the sections shown in Figure S4.
(TIF)

Figure S4 **HIV-1 M and N Vpus reduce the cell surface expression of tetherin.** Images show representative confocal acquisitions that are indicated in Figure S3. Shown are merged images of Vpu (blue), tetherin (red) and TGN (green). Tetherin was not located at the cell surface (membrane indicated by the arrows) in cells transfected with constructs expressing the NL4-3, YBF30 and EK505 YBF-TMD Vpus, as determined by microscopic examination and analysis of the Vpu, tetherin and TGN signal intensities throughout the cells. The regions utilized to generate the profile plots are indicated by the arrows.
(TIF)

Figure S5 **Colocalization of various Vpus with tetherin and the TGN.** (**A**) HeLa cells were transfected with Vpu expression constructs. At 48 hrs post-transfection the cells were fixed, stained for Vpu (blue), tetherin (red) and the TGN marker TGN46 (green) and examined by confocal microscopy. The bottom panel provides the merged overview and the upper panels show representative examples of the distribution of Vpu and tetherin in the TGN region. (**B, C**) The R^2^ values for the colocalization of Vpu with (B) the TGN and (C) tetherin indicate Pearson's correlation coefficient and were calculated for whole cells (n = 11–15) using Zen 2009 (Zeiss). Results were analyzed by unpaired 2-tailed t-test.
(TIF)

Figure S6 **Anti-tetherin activity of fusions between N- and CPZ-Vpus.** (**A**) Chimeras between HIV-1 N SJGddd and CK1.62 and SIVcpz*Ptt* LB7 and EK505 Vpu proteins. Dashes indicate gaps introduced to optimize the alignment. Expression of the Vpu constructs was confirmed by Western blotting (Fig. S2 and data not shown). (**B**) Effect of the indicated wild type and chimeric HIV-1 N and SIVcpz Vpu proteins on infectious virus release in the presence of tetherin. Curves represent the average infection values (n = 3) relative to those obtained in the absence of tetherin.
(TIF)

Figure S7 **Impact of lack of ß-TrCP activity on Vpu function.** (**A**) Infectious virus release from 293T cells transfected with a ΔVpu proviral NL4-3 construct (4 µg), a vector expressing the ß-TrCP1ΔFbox mutant (1 µg), and/or the indicated Vpu (1 µg) and tetherin (250 ng) expression constructs. Infectious virus was determined by triplicate infection of TZM-bl indicator cells and the results show averages (±SEM) derived from two independent experiments. (**B–D**) Lack of ß-TrCP activity impair Vpu-mediated down-modulation of tetherin and CD4 but not CD1d. Shown is the Vpu-dependent reduction of (**B**) tetherin, (**C**) CD4 and (**D**) CD1d surface expression in the presence or absence of the trans-dominant negative ß-TrCPΔFbox mutant. Shown are average values (±SD) from triplicate experiments.
(TIF)

Figure S8 **Recombination analysis of N1FR2011.** Bootstrap support values for the phylogenetic clustering of the N1FR2011 sequence with HIV-1 group N (green), HIV-1 group M (red), and SIVcpz*Ptt* EK505 (blue), the most closely related SIVcpz to HIV-1 group N, are shown. Values were obtained for a 200 bp window moved in 20 bp increments across an alignment of all available full-length HIV-1 group N sequences, SIVcpz EK505, and the Los Alamos HIV Sequence Database 2010 subtype reference sequences (www.hiv.lanl.gov/content/sequence/NEWALIGN/align.html). For no segment was the affinity of N1FR2011 with HIV-1 group M sequences supported at ≥70% (dashed line), indicating that it does not represent an intergroup (M/N) recombinant.
(TIF)

Figure S9 **The HIV-1 N1FR2011 N-Vpu is an effective tetherin antagonist.** Western blot analysis of HIV-1 particle release. 293T cells were cotransfected with a *vpu*-deleted NL4-3 proviral construct, with vectors expressing the indicated Vpu proteins and a plasmid expressing human tetherin (+) or an empty control vector (−). Cell and virion lysates were probed with an anti-capsid monoclonal antibody. A quantification of two independent Western Blots is shown in Figure 6G.
(TIF)

Figure S10 **Some putative functional domains or residues in the cytoplasmic part of Vpu are conserved in HIV-1 M and SIVcpz but not in HIV-1 N strains.** Frequency plots of amino acid residues in a putative YxK/RΦ motif, the DSGxxS ß-TrCP interaction site and a possible D/ExxxLL/I/V/M interaction site with adaptor protein complexes. Please note that in some cases the high sequence divergency of Vpu makes it difficult to accurately determine the presence of these sequence motifs.
(TIF)

Table S1 **Overview of HIV-1 N** ***vpu*** **alleles.** The table provides an overview on the origin of HIV-1 group N Vpu alleles.
(DOC)
