# Supplementary figures and images for: Human Tetherin Exerts Strong Selection Pressure on the HIV-1 Group N Vpu Protein
Source: PLoS Pathog. 2012 Dec 20;8(12):e1003093. doi: 10.1371/journal.ppat.1003093 (PMC3534379; doi:10.1371/journal.ppat.1003093)

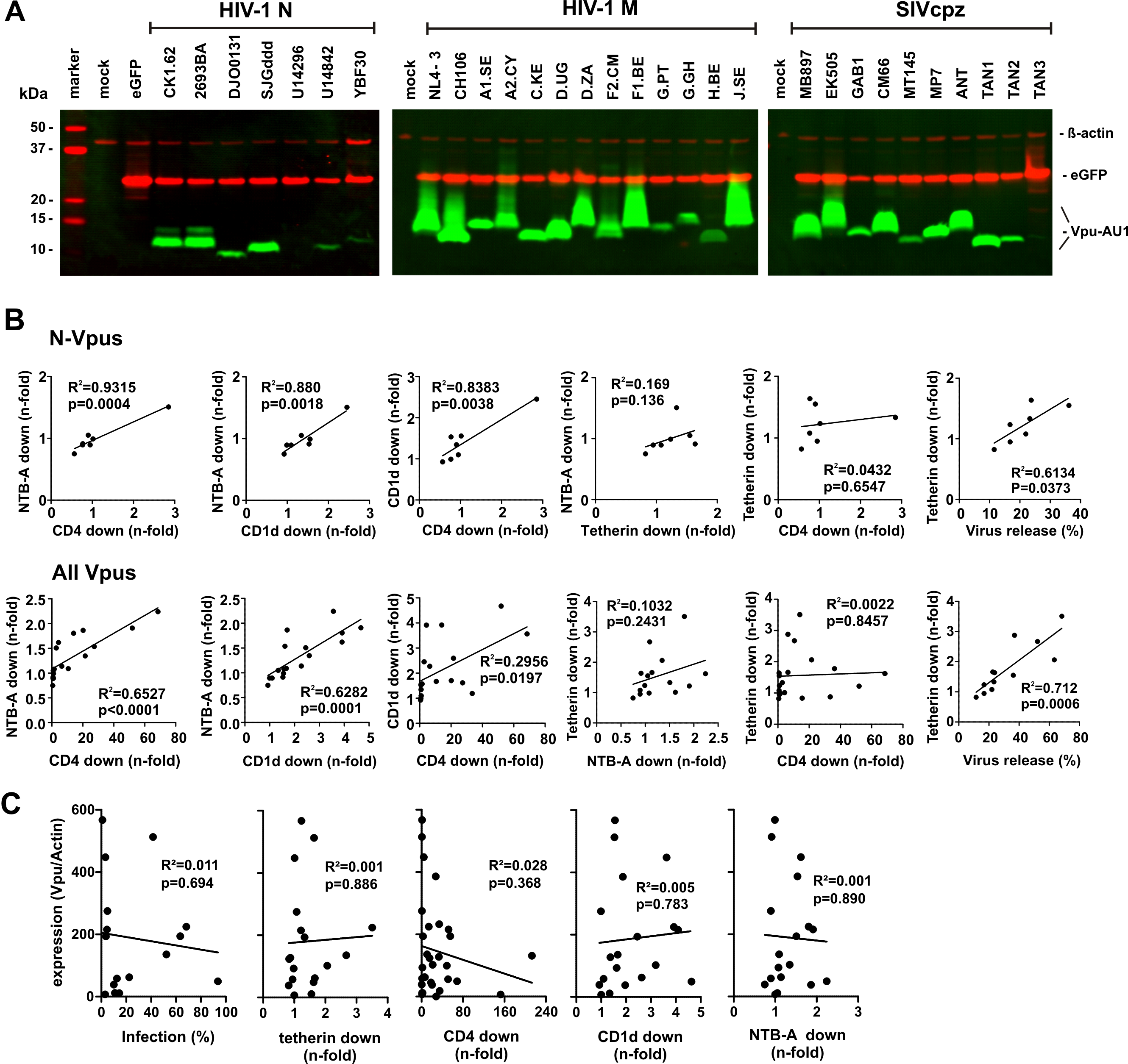

Supplement: Figure S1 — Vpu expression and correlation between various Vpu activities. (A) Expression of selected HIV-1 and SIV Vpu proteins. 293T cells were transfected with expression plasmids encoding the indicated AU1-tagged Vpus and eGFP. Mock transfected cells were used as negative controls; ß-Actin and eGFP expression levels were analyzed to control for loading and transfection efficiency, respectively. (B) Correlation between various Vpu activities. The upper panel shows results obtained for the seven N-Vpus and the lower panel shows data obtained for all HIV-1 and SIVcpz Vpus analyzed. (C) Vpu expression levels detected by immunoblot do not correlate with functional activity. Vpu expression levels were quantified from the Western blots shown in panel A and normalized to ß-actin. N-fold down specifies the reduction of mean fluorescence intensities obtained for the different markers in the presence of Vpu compared to those measured in cells transfected with the eGFP control construct. Viral release in the presence of tetherin is expressed as the percentage of viral infectivity in the absence of the restriction factor (100%). (TIF) [file ppat.1003093.s001.tif]

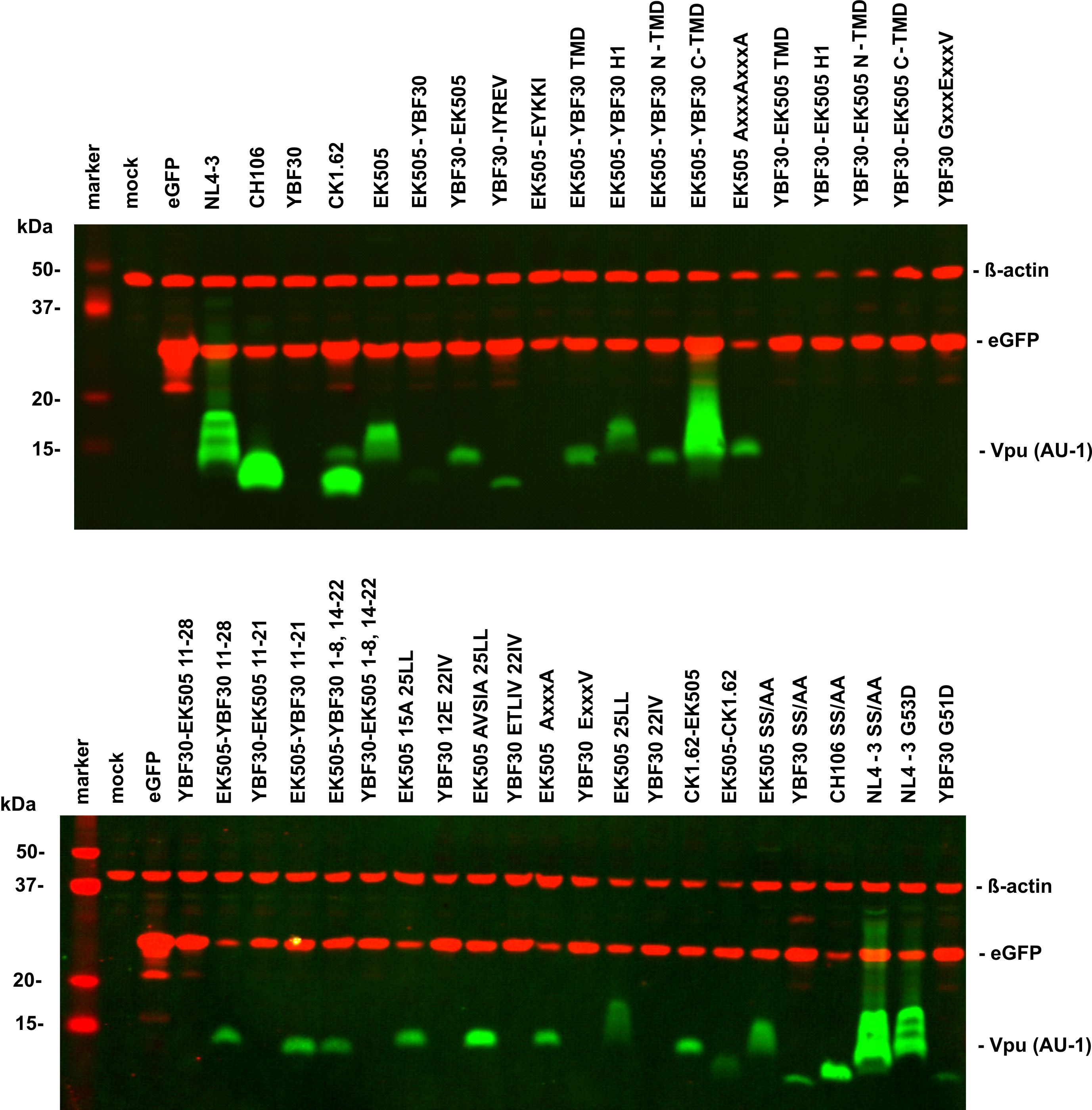

Supplement: Figure S2 — Expression of Vpu chimeras and mutants. Expression of the indicated wild-type, mutant and chimeric Vpu proteins was determined as described in the legend to Fig. S1. The amino acid sequences of the mutant Vpus are shown in Figs. 3A and 4A. Similar results were obtained in an independent experiment. (TIF) [file ppat.1003093.s002.tif]

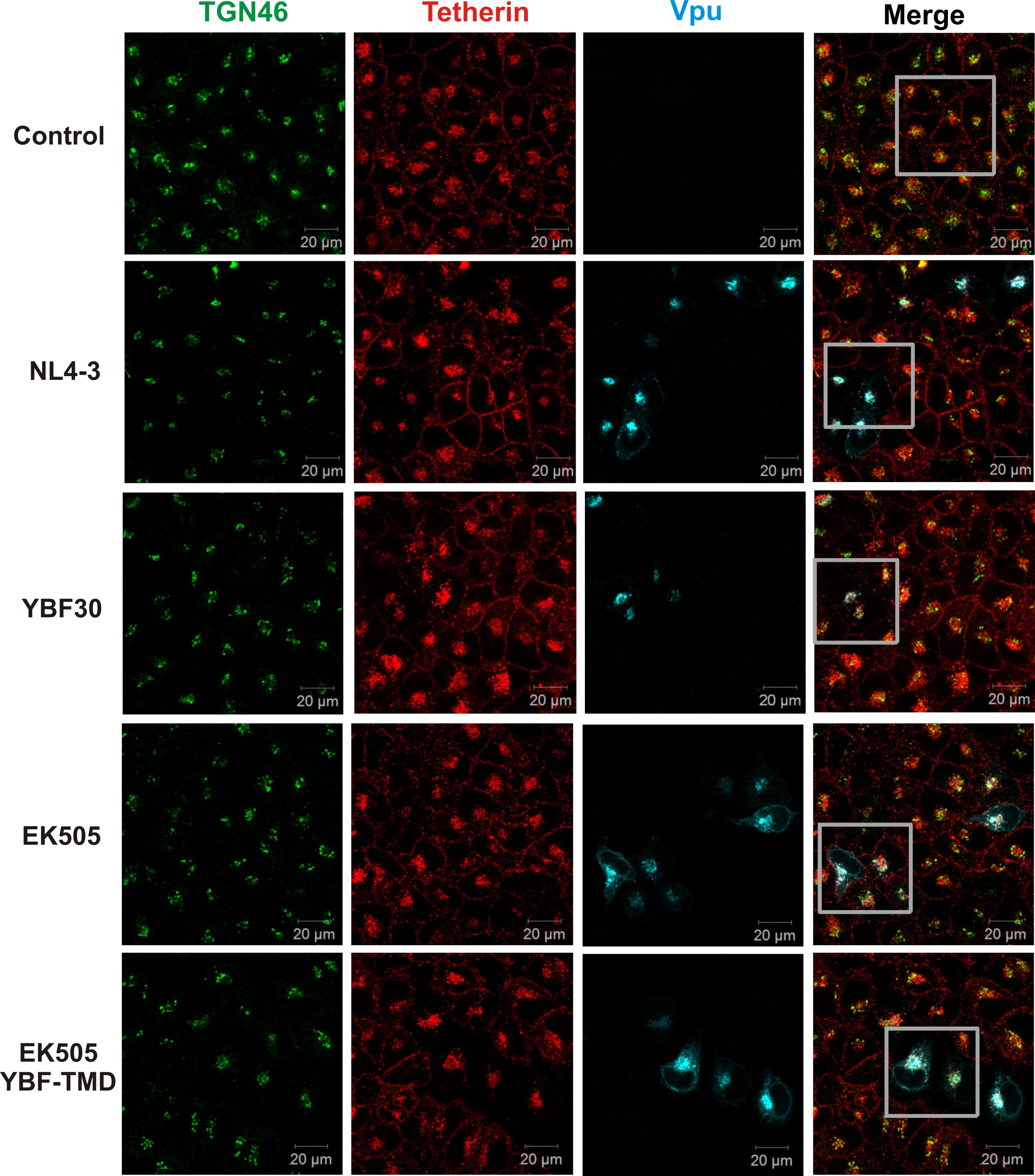

Supplement: Figure S3 — Overview on the cellular localization of tetherin and various Vpu proteins. Confocal immunofluorescence images of HeLa cells transfected with constructs expressing the indicated Vpu variants. Two days post-transfection, cells were fixed and permeabilized for intracellular staining of tetherin (red), Vpu (blue) and the TGN (green). Images show an overview on confocal acquisitions. The squares in the right panels indicate the sections shown in Figure S4. (TIF) [file ppat.1003093.s003.tif]

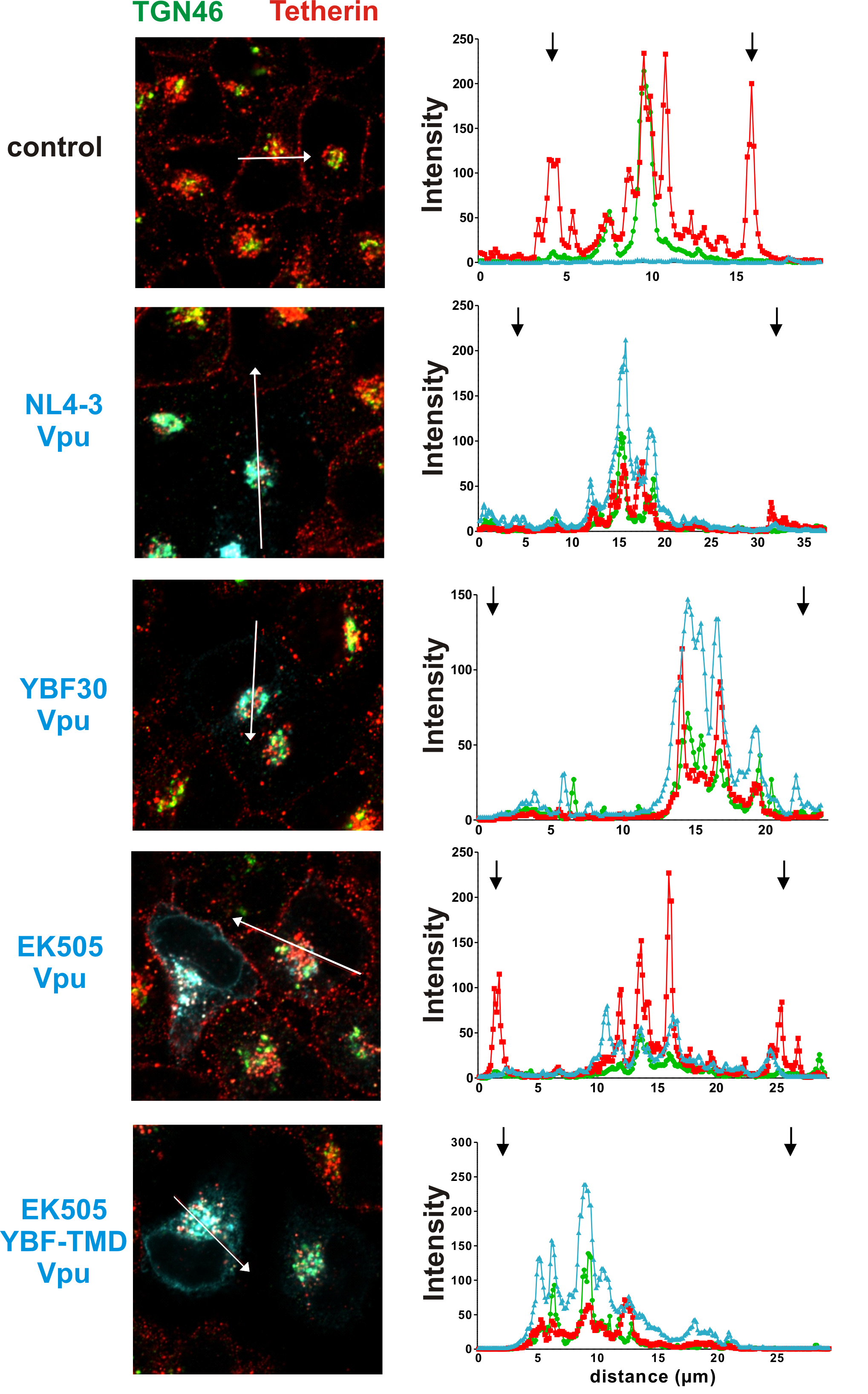

Supplement: Figure S4 — HIV-1 M and N Vpus reduce the cell surface expression of tetherin. Images show representative confocal acquisitions that are indicated in Figure S3. Shown are merged images of Vpu (blue), tetherin (red) and TGN (green). Tetherin was not located at the cell surface (membrane indicated by the arrows) in cells transfected with constructs expressing the NL4-3, YBF30 and EK505 YBF-TMD Vpus, as determined by microscopic examination and analysis of the Vpu, tetherin and TGN signal intensities throughout the cells. The regions utilized to generate the profile plots are indicated by the arrows. (TIF) [file ppat.1003093.s004.tif]

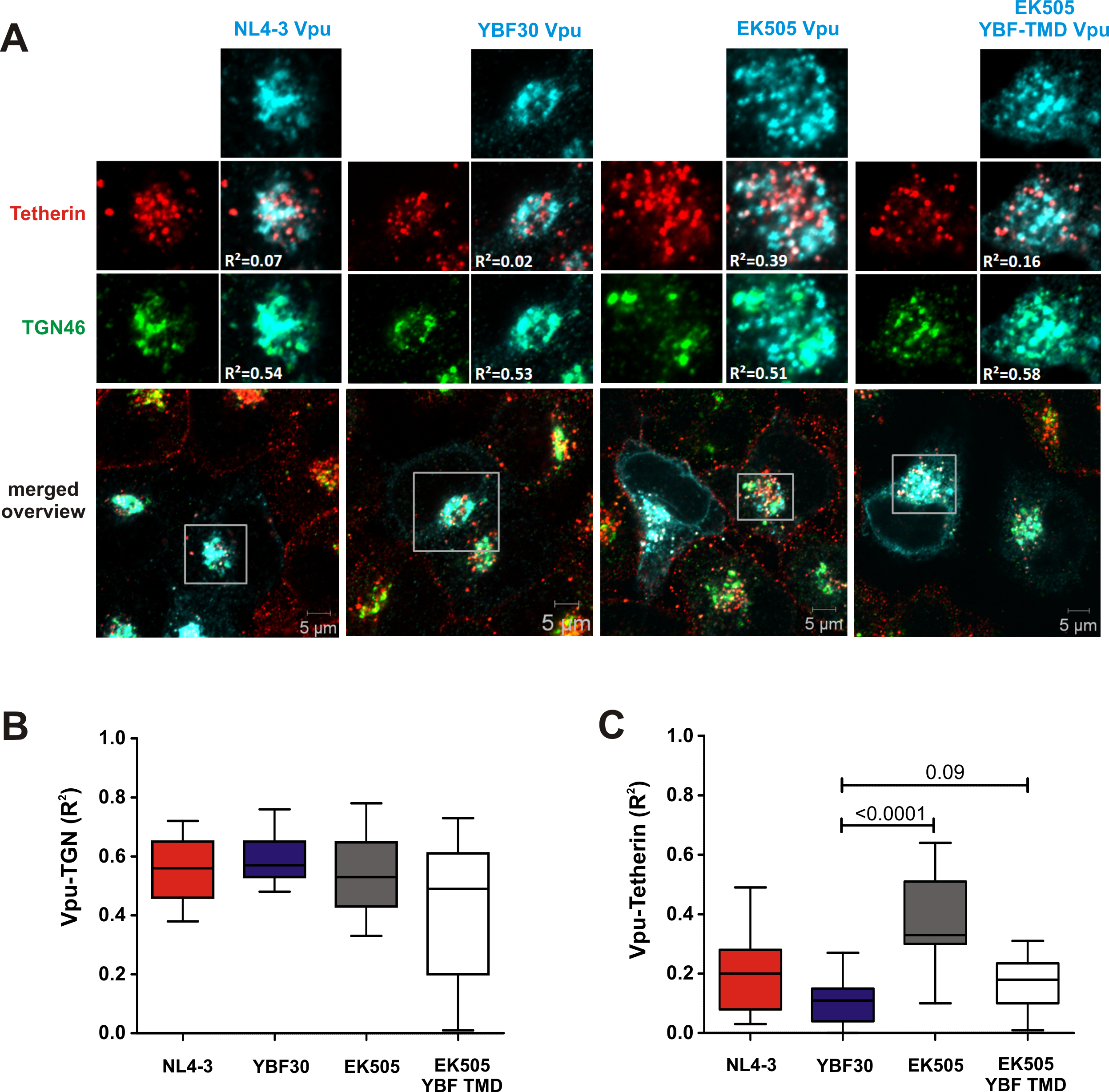

Supplement: Figure S5 — Colocalization of various Vpus with tetherin and the TGN. (A) HeLa cells were transfected with Vpu expression constructs. At 48 hrs post-transfection the cells were fixed, stained for Vpu (blue), tetherin (red) and the TGN marker TGN46 (green) and examined by confocal microscopy. The bottom panel provides the merged overview and the upper panels show representative examples of the distribution of Vpu and tetherin in the TGN region. (B, C) The R2 values for the colocalization of Vpu with (B) the TGN and (C) tetherin indicate Pearson's correlation coefficient and were calculated for whole cells (n = 11–15) using Zen 2009 (Zeiss). Results were analyzed by unpaired 2-tailed t-test. (TIF) [file ppat.1003093.s005.tif]

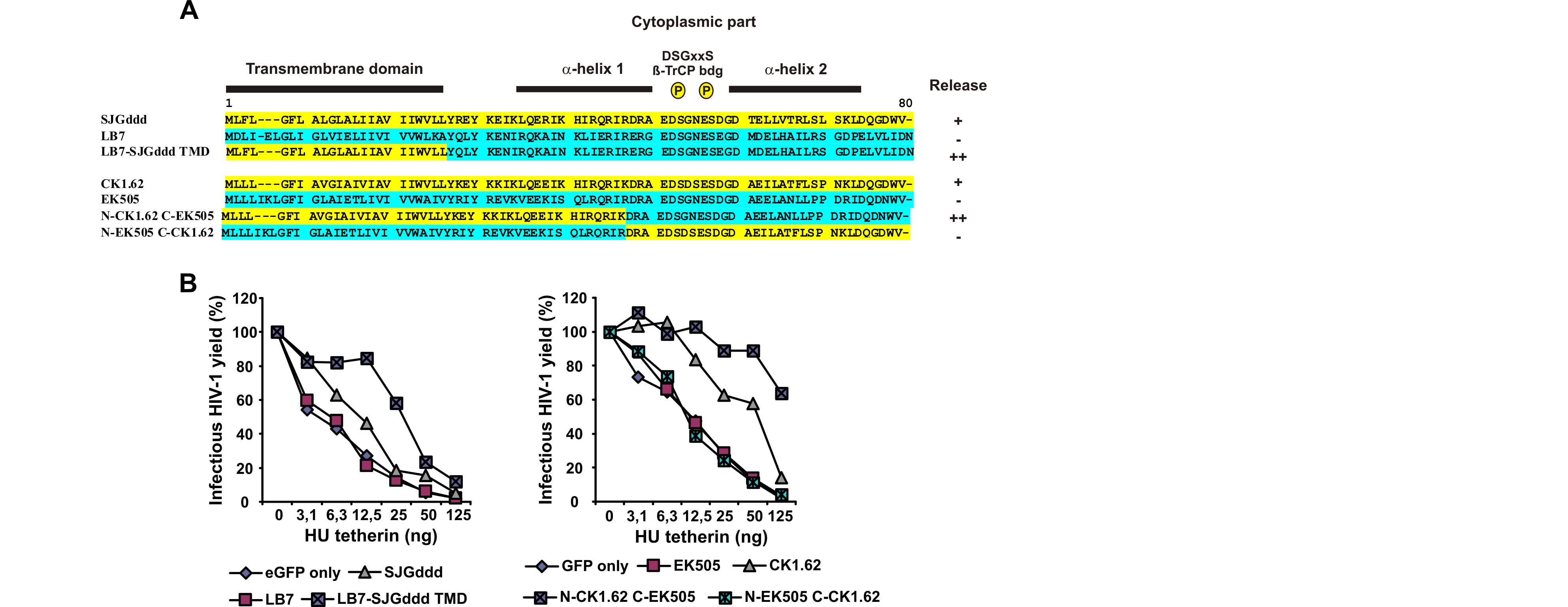

Supplement: Figure S6 — Anti-tetherin activity of fusions between N- and CPZ-Vpus. (A) Chimeras between HIV-1 N SJGddd and CK1.62 and SIVcpzPtt LB7 and EK505 Vpu proteins. Dashes indicate gaps introduced to optimize the alignment. Expression of the Vpu constructs was confirmed by Western blotting (Fig. S2 and data not shown). (B) Effect of the indicated wild type and chimeric HIV-1 N and SIVcpz Vpu proteins on infectious virus release in the presence of tetherin. Curves represent the average infection values (n = 3) relative to those obtained in the absence of tetherin. (TIF) [file ppat.1003093.s006.tif]

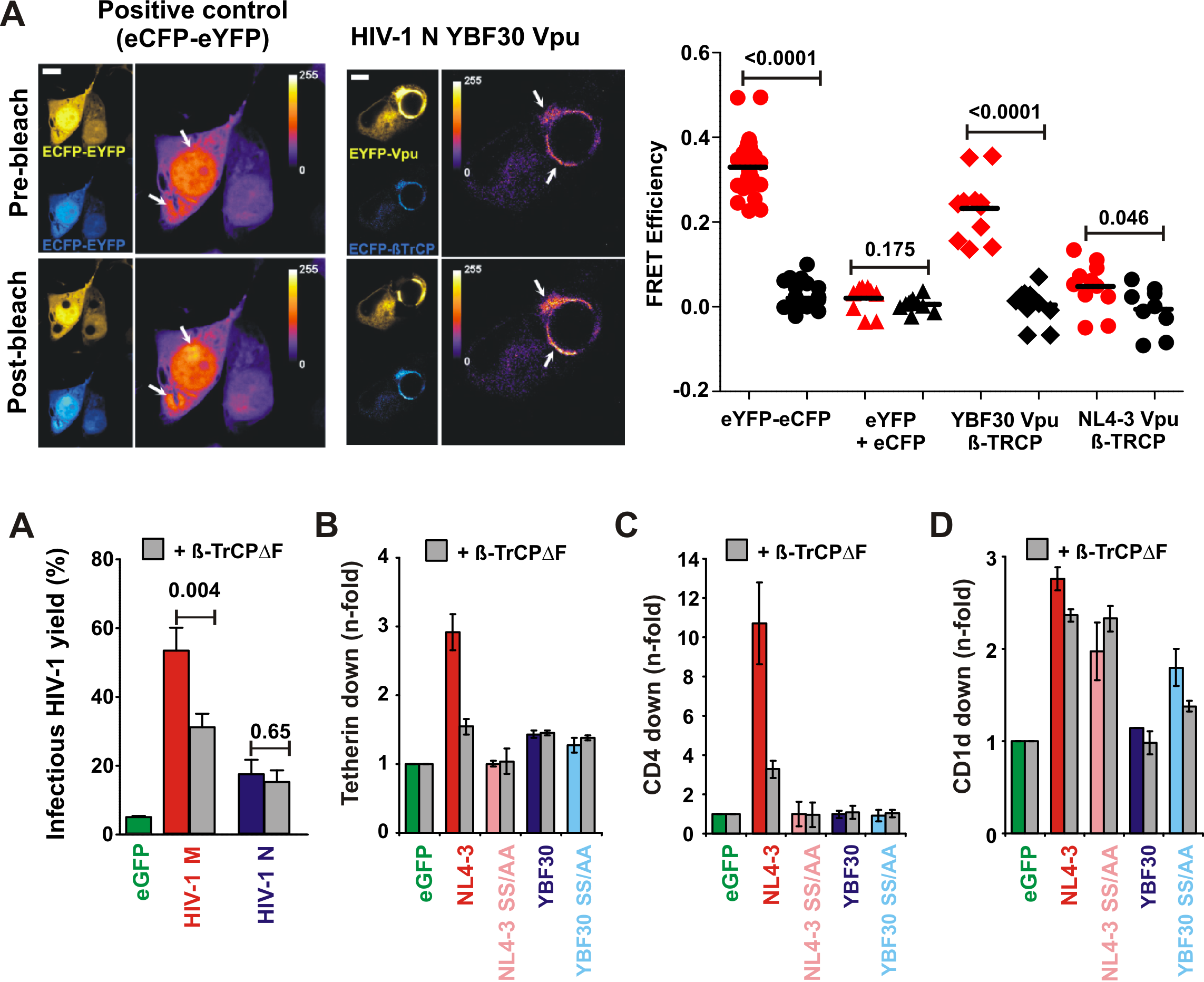

Supplement: Figure S7 — Impact of lack of ß-TrCP activity on Vpu function. (A) Infectious virus release from 293T cells transfected with a ΔVpu proviral NL4-3 construct (4 µg), a vector expressing the ß-TrCP1ΔFbox mutant (1 µg), and/or the indicated Vpu (1 µg) and tetherin (250 ng) expression constructs. Infectious virus was determined by triplicate infection of TZM-bl indicator cells and the results show averages (±SEM) derived from two independent experiments. (B–D) Lack of ß-TrCP activity impair Vpu-mediated down-modulation of tetherin and CD4 but not CD1d. Shown is the Vpu-dependent reduction of (B) tetherin, (C) CD4 and (D) CD1d surface expression in the presence or absence of the trans-dominant negative ß-TrCPΔFbox mutant. Shown are average values (±SD) from triplicate experiments. (TIF) [file ppat.1003093.s007.tif]

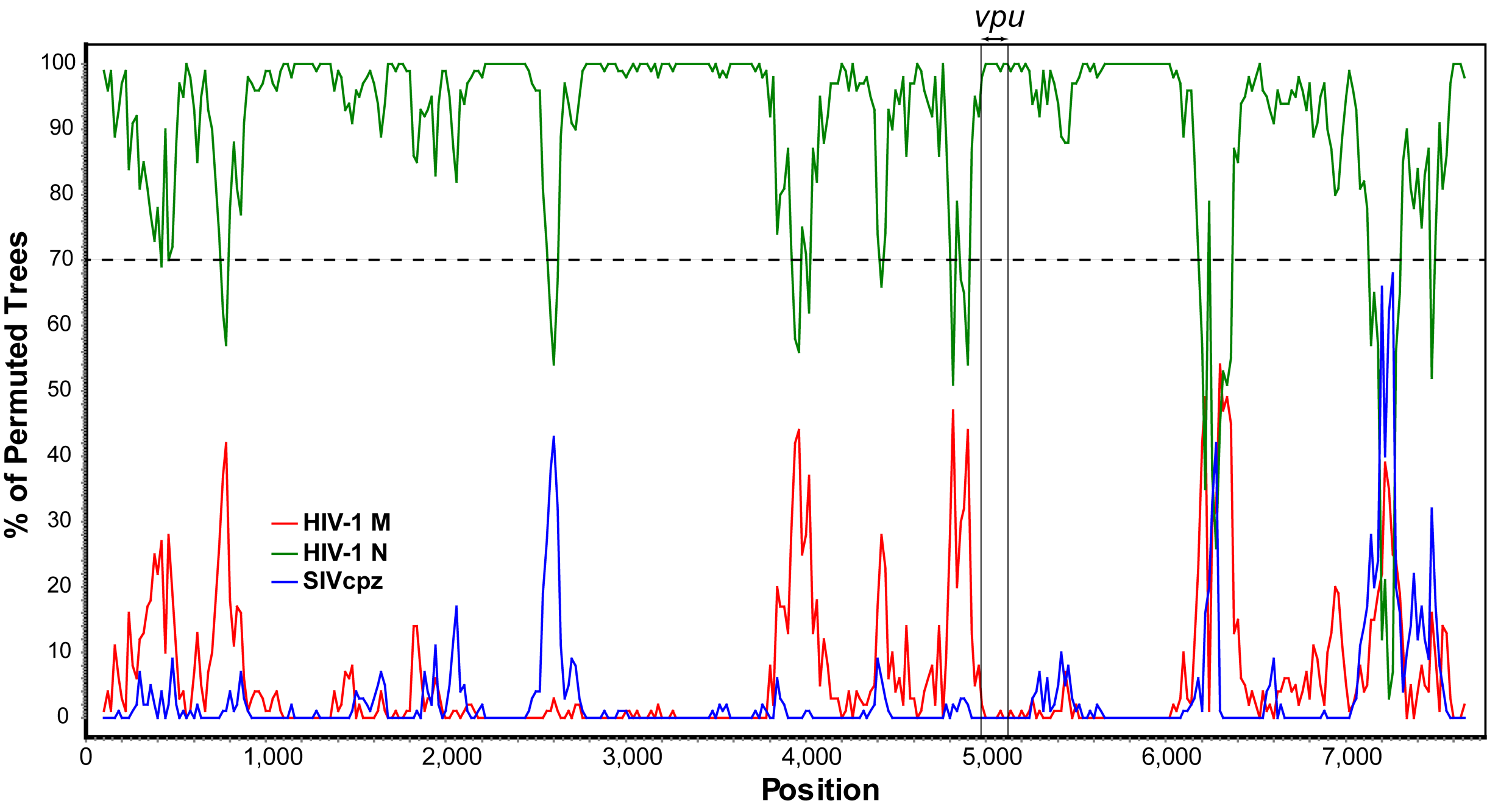

Supplement: Figure S8 — Recombination analysis of N1FR2011. Bootstrap support values for the phylogenetic clustering of the N1FR2011 sequence with HIV-1 group N (green), HIV-1 group M (red), and SIVcpzPtt EK505 (blue), the most closely related SIVcpz to HIV-1 group N, are shown. Values were obtained for a 200 bp window moved in 20 bp increments across an alignment of all available full-length HIV-1 group N sequences, SIVcpz EK505, and the Los Alamos HIV Sequence Database 2010 subtype reference sequences (www.hiv.lanl.gov/content/sequence/NEWALIGN/align.html). For no segment was the affinity of N1FR2011 with HIV-1 group M sequences supported at ≥70% (dashed line), indicating that it does not represent an intergroup (M/N) recombinant. (TIF) [file ppat.1003093.s008.tif]

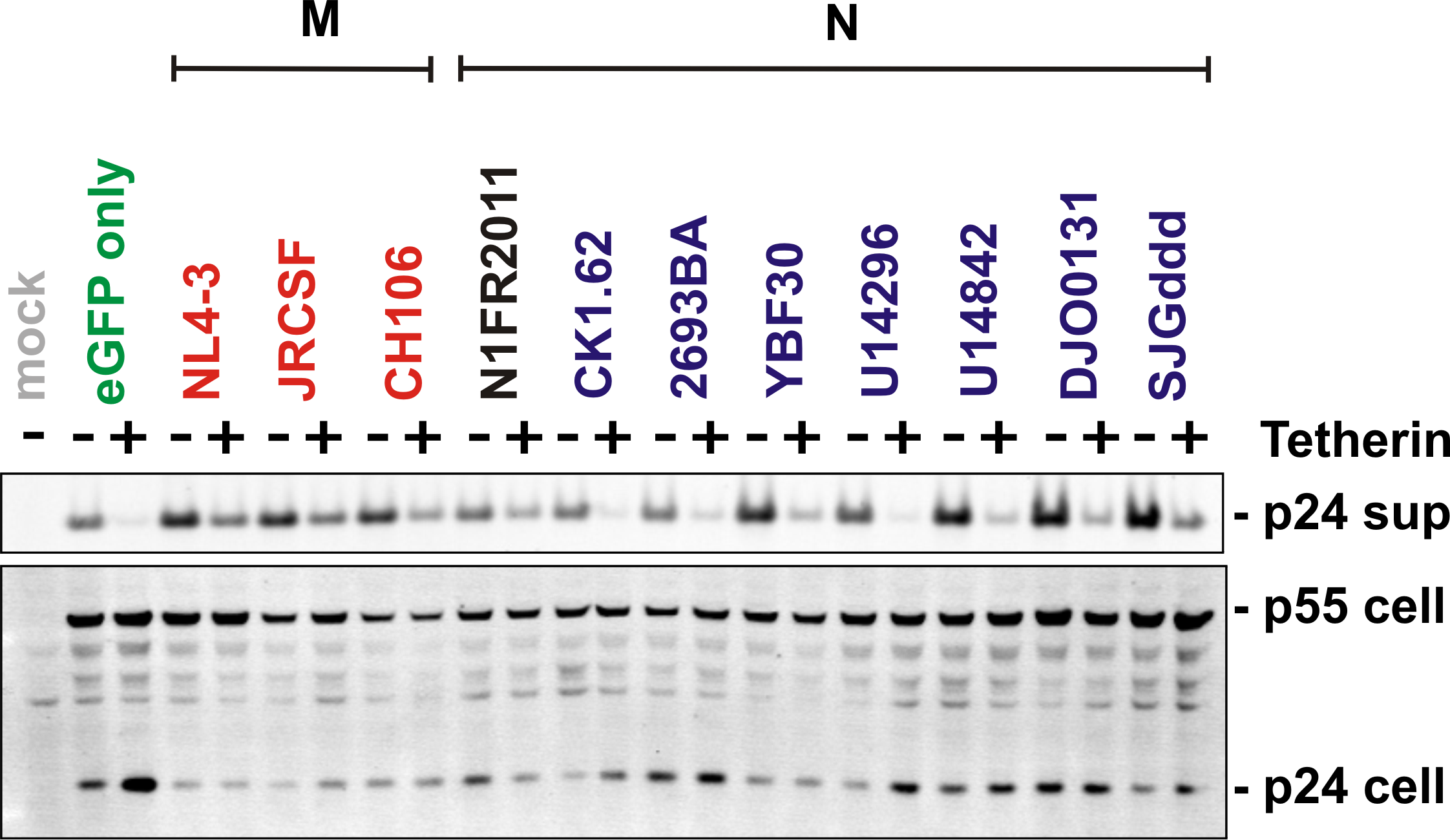

Supplement: Figure S9 — The HIV-1 N1FR2011 N-Vpu is an effective tetherin antagonist. Western blot analysis of HIV-1 particle release. 293T cells were cotransfected with a vpu-deleted NL4-3 proviral construct, with vectors expressing the indicated Vpu proteins and a plasmid expressing human tetherin (+) or an empty control vector (−). Cell and virion lysates were probed with an anti-capsid monoclonal antibody. A quantification of two independent Western Blots is shown in Figure 6G. (TIF) [file ppat.1003093.s009.tif]

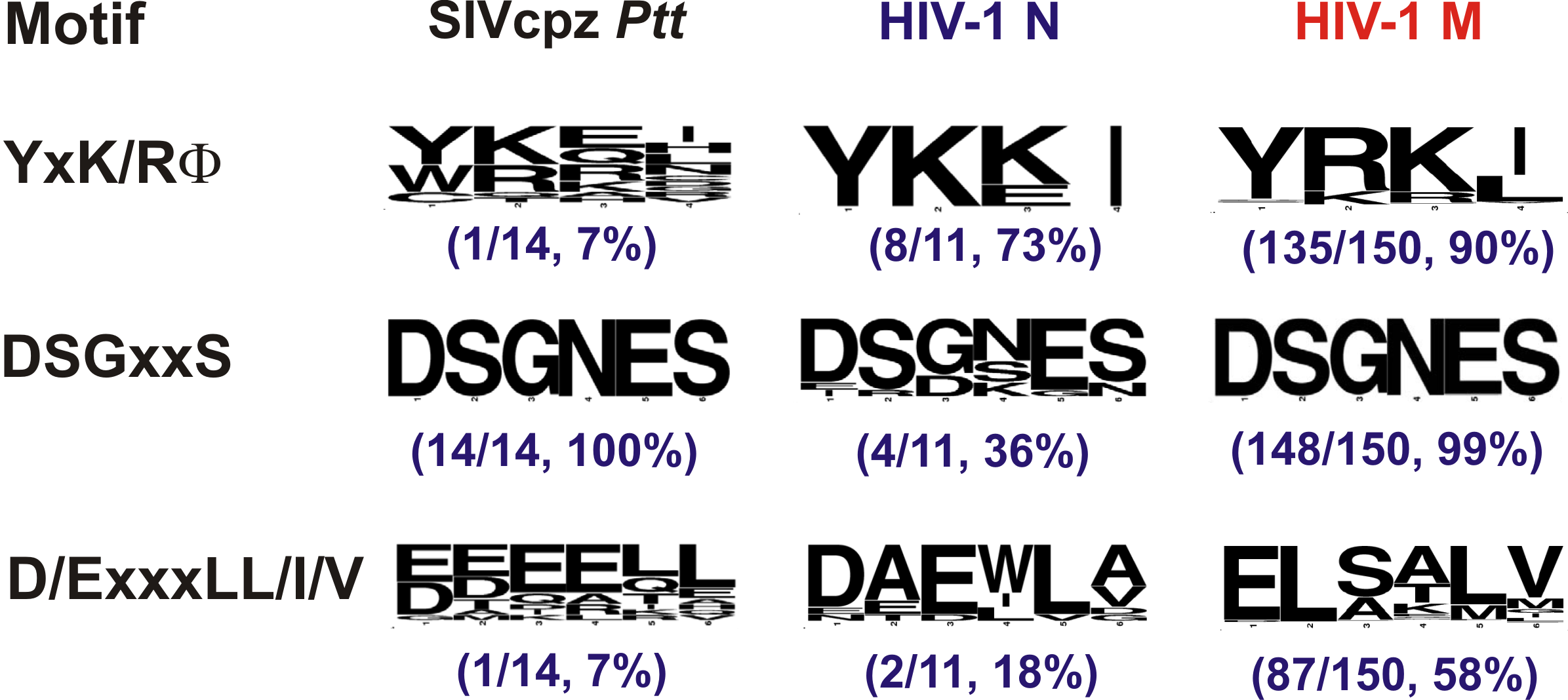

Supplement: Figure S10 — Some putative functional domains or residues in the cytoplasmic part of Vpu are conserved in HIV-1 M and SIVcpz but not in HIV-1 N strains. Frequency plots of amino acid residues in a putative YxK/RΦ motif, the DSGxxS ß-TrCP interaction site and a possible D/ExxxLL/I/V/M interaction site with adaptor protein complexes. Please note that in some cases the high sequence divergency of Vpu makes it difficult to accurately determine the presence of these sequence motifs. (TIF) [file ppat.1003093.s010.tif]
