# Supplementary material for: Human Tetherin Exerts Strong Selection Pressure on the HIV-1 Group N Vpu Protein
Source: PLoS Pathog. 2012 Dec 20;8(12):e1003093. doi: 10.1371/journal.ppat.1003093 (PMC3534379; doi:10.1371/journal.ppat.1003093)
Supplement: Table S1 — Overview of HIV-1 N vpu alleles. The table provides an overview on the origin of HIV-1 group N Vpu alleles. (DOC) [file ppat.1003093.s011.doc]

**Table S1.** Overview of HIV-1 N *vpu* alleles.

Clone DSGxxS Source Year of origin Patient AIDS/symptoms Genbank Reference

motif sampling age/sex accession #

YBF30* Yes PBMC coculture 1995 Cameroon 40 W AIDS, died in 1997 AJ006022 [5]

YBF106 No uncultured PBMC 1997 Cameroon 51 M AIDS, died in 1998 AJ271370 [4]

2693BA* No buffy coat 2001 Cameroon n.k. n.k. GQ925950 [19]

CK1.62* No buffy coat 2002 Cameroon n.k. n.k. GQ925951 [19]

DJO0131* No plasma 2002 Cameroon n.k. n.k. [AY532635](http://www.hiv.lanl.gov/components/sequence/HIV/asearch/query_one.comp?se_id=159509) [2]

SJGddd* Yes plasma 2002 Cameroon n.k. n.k. [GQ324959](http://www.hiv.lanl.gov/components/sequence/HIV/asearch/query_one.comp?se_id=344852) [6]

1131-03b No plasma/whole blood 2004 Cameroon 44 M CD4+ T cells 92/µl^+^ DQ017383 [48]

1015-04 Yes plasma/whole blood 2004 Cameroon 48 W asymptomatic DQ017382 [48]

U14296* No blood 2006 Cameroon 47 W n.k. [GQ324962](http://www.hiv.lanl.gov/components/sequence/HIV/asearch/query_one.comp?se_id=344849) [6]

U14842* No blood 2006 Cameroon 48 M n.k. [GQ324958](http://www.hiv.lanl.gov/components/sequence/HIV/asearch/query_one.comp?se_id=344853) [6]

N1FR2011* Yes Plasma RNA 2011 Togo/France 57 M early CD4 decline JN572926 [3]

* *vpu* alleles functionally analyzed; ^+^ before antiretroviral treatment; n.k., not known.
